# Radial Glial Neural Progenitors Regulate Nascent Brain Vascular Network Stabilization Via Inhibition of Wnt Signaling

**DOI:** 10.1371/journal.pbio.1001469

**Published:** 2013-01-22

**Authors:** Shang Ma, Hyo Jun Kwon, Heidi Johng, Keling Zang, Zhen Huang

**Affiliations:** 1 Departments of Neuroscience and Neurology, University of Wisconsin–Madison, Madison, Wisconsin, United States of America; 2 Graduate Program in Cellular and Molecular Biology, University of Wisconsin–Madison, Madison, Wisconsin, United States of America; 3 Neuroscience Training Program, University of Wisconsin–Madison, Madison, Wisconsin, United States of America; 4 Department of Physiology, University of California–San Francisco, San Francisco, California, United States of America; Stanford University School of Medicine, United States of America

## Abstract

The cerebral cortex performs complex cognitive functions at the expense of tremendous energy consumption. Blood vessels in the brain are known to form stereotypic patterns that facilitate efficient oxygen and nutrient delivery. Yet little is known about how vessel development in the brain is normally regulated. Radial glial neural progenitors are well known for their central role in orchestrating brain neurogenesis. Here we show that, in the late embryonic cortex, radial glial neural progenitors also play a key role in brain angiogenesis, by interacting with nascent blood vessels and regulating vessel stabilization via modulation of canonical Wnt signaling. We find that ablation of radial glia results in vessel regression, concomitant with ectopic activation of Wnt signaling in endothelial cells. Direct activation of Wnt signaling also results in similar vessel regression, while attenuation of Wnt signaling substantially suppresses regression. Radial glial ablation and ectopic Wnt pathway activation leads to elevated endothelial expression of matrix metalloproteinases, while inhibition of metalloproteinase activity significantly suppresses vessel regression. These results thus reveal a previously unrecognized role of radial glial progenitors in stabilizing nascent brain vascular network and provide novel insights into the molecular cascades through which target neural tissues regulate vessel stabilization and patterning during development and throughout life.

## Abstract 2

Radial glial cells, which are neural stem cells well known for their role in neurogenesis, also play an unexpected role in stabilizing nascent blood vessels in the brain.

## Author Summary

The brain is an energy-intensive organ that consumes about 10 times as much energy per unit volume as the rest of the body. It therefore requires a highly efficient vascular network for oxygen and nutrient delivery, and as a result compromises in blood vessel networks influence a wide array of brain diseases. Our current understanding is that brain-specific neural cell types are involved in shaping its vascular network, but unfortunately little is known about the cellular or molecular mechanisms involved. Using a mouse genetic model, we have found that radial glial cells, a stem cell type well known for its fundamental role in neural circuit formation, also play an unexpected role in brain vessel development. We find that radial glial cells are essential for the stabilization of newly formed blood vessels in the late embryonic brain, and do so in large part through down-regulating canonical Wnt signaling in endothelial cells (which line the interior surface of blood vessels). These findings provide new insight into how new vessels in the brain are normally stabilized and how this process may be compromised and contribute to diseases.

## Introduction

The brain consumes approximately10 times as much energy per unit volume as the rest of the body and thus requires a highly efficient vascular network for oxygen and nutrient delivery as well as waste disposal. Cortical blood vessels display a highly complex and hierarchical pattern [1],[2], of which a most striking feature is the regularity by which large vessels penetrate the cortex from the pia at right angles. These vessels then give off branches and capillaries at various depths, yielding an intricate network. Such stereotypic organizations provide a unique opportunity for understanding how target-specific cell types and signals regulate vascular network formation and patterning and coordinate neural and vascular function during development and throughout life.

Vascular patterning, in principle, may be regulated by guided growth as well as selective stabilization, as is probably best demonstrated in the formation of another cellular network, the neural circuitry 3,4. Indeed, several axon guidance cues have been identified that direct vessel growth [5],[6]. For example, semaphorins and netrins have been found to restrict vessel growth to intersomitic regions during embryogenesis [7],[8], while peripheral nerves appear to determine patterns of vessel branching and differentiation, in part through local secretion of vascular endothelial growth factor (VEGF) [9]. In contrast, little is known about how target neural tissues regulate the later step of vessel stabilization.

Neural cells have long been known to play a key role in vessel differentiation in the central nervous system (CNS). In the developing retina, endothelial cells (ECs) follow a meshwork laid down by astrocytes [10]. Astrocytes also appear to induce CNS-specific EC differentiation [11]. In the embryonic cerebral cortex, astrocytes are known to be largely absent. However, there is another cell type with notable similarities [12]–[14]. These are the radial glia, primary neural progenitors of the developing cortex that also closely interact with growing vessels [15],[16]. Moreover, several neural-specific mutations also result in compromised brain vessel development [17]–[20], which further supports a role of neural cells in regulating CNS angiogenesis.

Studies have shown that, both inside and outside the nervous system, canonical Wnt signaling is a major pathway that regulates several steps of blood vessel development, including initial neural-tube vessel ingression, retinal vessel stabilization, intersomitic vessel remodeling, and hyaloid vessel regression [19],[21]–[25]. Interestingly, during early CNS vessel development, Wnt signaling from neural progenitors has been found to be essential for initial vessel ingression from outside into the neural tube [19],[23]. By contrast, in the eye, Wnt signaling from macrophages induces hyaloid vessel regression [21],[22]. This suggests that Wnt signaling can have distinct, and even opposite, effects at different stages of angiogenesis and/or in different tissues. Moreover, constitutively active Wnt signaling also leads to compromised vessel development throughout the embryo [25]. This indicates that the level of Wnt signaling is also crucial for normal angiogenesis. Thus, these results suggest that canonical Wnt signaling is tightly and dynamically regulated and plays a multifaceted role during blood vessel development.

Here we investigate the role of radial glial progenitors in vessel stabilization during late embryonic corticogenesis, following the formation of a primitive vascular network. We find that ablation of radial glia at this stage results in vessel regression, which suggests a role of radial glia in vessel stabilization. Vessel regression under these conditions is closely linked to ectopic activation in ECs of canonical Wnt signaling and is mimicked by activation, but substantially suppressed by attenuation, of Wnt pathway activity. This indicates that radial glia control vessel stabilization, in large part, through inhibition of Wnt signaling. Furthermore, we find that radial glia inhibits EC Wnt signaling at E15.5 but not E13.5. This indicates a stage-specific inhibition of EC Wnt signaling by radial glia. Lastly, we find that ablation of radial glia and activation of Wnt signaling each leads to elevated expression in ECs of matrix metalloproteinases, while attenuation of metalloproteinase activity substantially suppresses vessel regression. This suggests that vessel stabilization by radial glia may be in part mediated through inhibition of metalloproteinase expression. Together, these results reveal novel insights into the molecular cascade(s) through which neural progenitors regulate vessel stabilization and patterning during brain development.

## Results

### Vessel Growth in the Embryonic Cerebral Cortex

**Figure 1:**
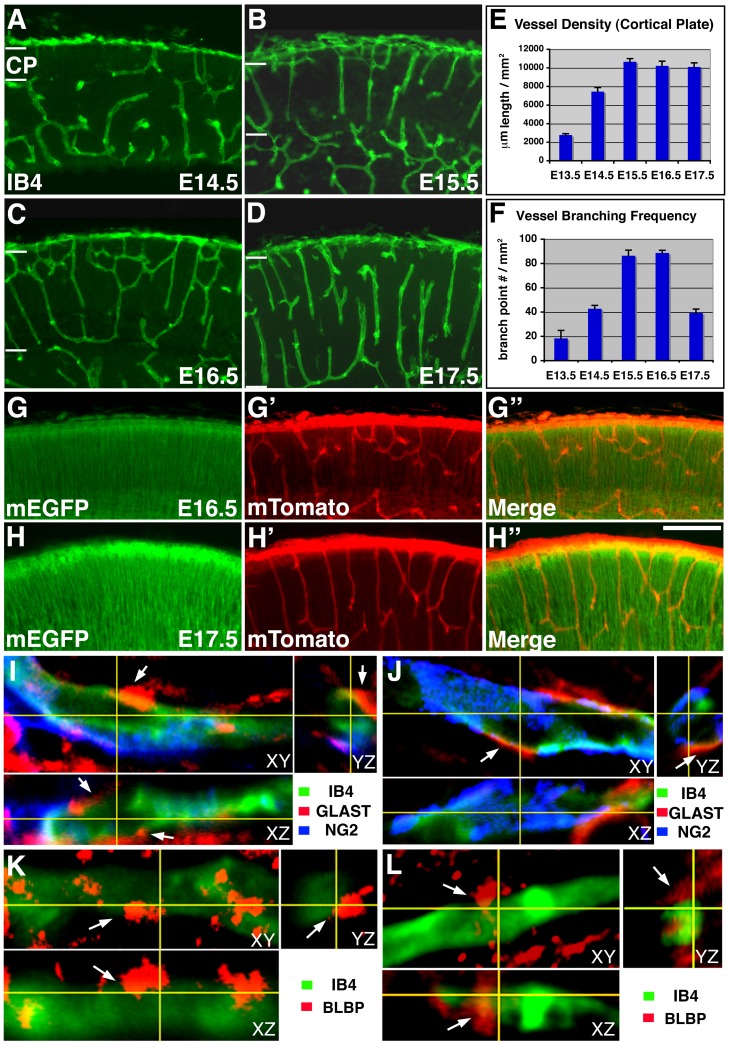
Vascular development in the mouse embryonic cortex. (A–D) Patterns of vessel growth from E14.5 to E17.5. IB4 (in green) was used to stain growing vessels in the cortical plate (CP, outlined by pairs of white bars in A–D). A small number of vessels are observed at E14.5 (A), while increased numbers are observed at E15.5 (B) and E16.5 (C). By E17.5, the most prominent vessels run in the vertical orientation (D). (E–F) Quantification of vessel density (E) and branching frequency (F) in the cortical plate. Vessel branching frequency increases from E13.5 and peaks at E15.5 and E16.5. Subsequently, it drops dramatically at E17.5. By contrast, vessel density remains relatively stable after E15.5. (G–H″) Patterns of cortical plate blood vessels as visualized using an *mTomato/mEGFP* reporter, which, when driven by the neural-specific *nestin-cre*, expresses mEGFP (in green) in radial glia but mTomato (in red) in vessels at both E16.5 (G–G″) and E17.5 (H–H″). In contrast to E16.5, the vast majorities of vessels run vertically at E17.5. (I–J) Direct interactions between radial glia and ECs at E16.5 by 3-D reconstruction. ECs, pericytes, and radial glia were labeled with IB4 (in green) and NG2 (in blue) and GLAST (in red) antibodies, respectively. Cross-sections showed direct interactions between ECs and radial glia (arrows) despite significant pericyte coverage. (K–L) Direct interactions between radial glia and ECs were also observed at E16.5 by BLBP labeling of radial glia (in red). ECs were labeled by IB4 (in green). Cross-sections showed direct interactions between ECs and radial glia (arrows). Scale bar (in H″): 100 µm for (A–D) and (G–H″).

Previous studies have documented bursts of vessel sprouting at different depths in the postnatal rat cortex [26]. To determine how vessel growth in the prenatal murine cortex is regulated, we systematically examined isolectin B4 (IB4) staining from embryonic day12.5 (E12.5) to postnatal day 0 (P0) (Figure 1A–D, and unpublished data). We found that vessels from the pial perineural vascular plexus first invade the cortical plate around E12.5 (unpublished data). By E14.5, a significant density of vessels can be observed in the cortical plate (Figure 1A). As the cortical plate expands, vessel density increases at E15.5 (Figure 1B). Subsequently, at E16.5, a primitive vascular network begins to appear (Figure 1C). By E17.5, prominent vertically oriented vessels are observed (Figure 1D). Quantification revealed that vessel density in the cortical plate first rises sharply from E13.5 to E15.5 but stays relatively stable after E15.5 (Figure 1E). Concomitantly, vessel branch point frequency increases substantially from E13.5 to E15.5 and E16.5 (Figure 1F), suggesting sprouting as a major mode of growth during this early period. By E17.5, however, although vessel density remains comparable to those of E15.5 and E16.5 (Figure 1E), branch point frequency becomes dramatically lower than those of the earlier stages (Figure 1F). This suggests that most of the vessel growth from E16.5 to E17.5 likely results from elongation of existing vessels.

At E17.5, the vast majority of cortical plate vessels are aligned vertically (Figure 1D), in contrast to E16.5, when frequent horizontal branches are observed (Figure 1C). These changes suggest preferential elongation and stabilization of vertically oriented vessels and/or elimination of horizontal branches. Indeed, using an *mTomato/mEGFP* dual fluorescent *cre* reporter [27], which, under the control of a neural specific *nestin-cre* [28],[29], expresses mEGFP in targeted neural precursors and mTomato in unrecombined vascular cells, we also observed similar changes in the pattern of cortical plate vessels (Figure 1G–H″). At E16.5, we found that mTomato positive vessels show significant lateral branches (Figure 1G′). By contrast, at E17.5, the vast majority of vessels appear oriented vertically, with few lateral branches (Figure 1H′). Quantification of IB4 positive vessels also showed that there is not only a decrease in the frequency of vessel branch points from E16.5 to E17.5 (Figure 1F), but also a reduction in the absolute number of branch points over the entire cortical plate during this period (E16.5, 26±0.6; E17.5, 17±1.8; *p*<0.004, *n* = 3). Thus, vessel development in the embryonic cortical plate appears to go through an initial phase of sprouting, followed by remodeling during which vertically oriented vessels are stabilized.

The vertical orientation of cortical plate vessels at E17.5 is reminiscent of that of radial glial fibers. Previously, close interactions have been observed between blood vessels and intermediate progenitors in the subventricular zone [30]. To examine the interactions between blood vessels and radial glial fibers in the cortical plate at these late embryonic stages, we employed triple labeling and three-dimensional reconstruction to examine, simultaneously, the spatial relationship between radial glia, ECs, and pericytes at E16.5 (Figure 1I–J). We found that, despite significant coverage of blood vessels by percicytes at this stage, there is a similar proportion of EC surface areas that are exposed and through which ECs physically interact with radial glial fibers. Indeed, radial glia and pericytes frequently appear to interdigitate in their interactions with ECs (see also Movies S1 and S2). Similar direct interactions between radial glia and ECs were also observed when we used an anti–brain lipid binding protein (BLBP) antibody to label radial glia (Figure 1K–L). These findings suggest a potential role of radial glia in regulating vessel development at this stage. They are also consistent with previous electron microscopy data showing that ECs directly interact with astrocytes in the postnatal cortex [31]. In addition, in the late embryonic cortex of *β1 integrin/emx1-cre* mutants where radial glial fibers sometimes run obliquely, we also observed blood vessels running at the same angle (unpublished data). This further suggests a role of radial glia in regulating blood vessel development at this stage. Thus, these findings altogether raise the possibility that radial glia may play a role in regulating cortical plate vessel stabilization during late embryogenesis.

### Ablation of Radial Glial Progenitors by Cell Cycle Blockade During Late Embryogenesis

Radial glia constitute a major population of neural progenitors in the embryonic cortex, the rest being intermediate progenitors as well as a very small number of outer radial glia-like progenitors [12],[13]. Thus, we reasoned that neural lineage-specific blockade of cell cycle progression during late embryogenesis may allow relatively selective ablation of radial glia and an evaluation of their role in cortical vessel stabilization. To this end, we employed a conditional knockout allele we generated of *orc3*, a gene encoding a core subunit of origin recognition complex (ORC), a complex composed of Orc1–6, of which all are essential for DNA replication [32]. We targeted *orc3* locus by floxing exons 5–7, creating an early truncation (Figure S1A–C). We found that homozygotes of the floxed *orc3* allele are viable and fertile, without obvious phenotypes, while germline deletion of *orc3* results in early embryonic lethality. We also confirmed that Orc3 protein is lost from mutant cortices (Figure S1D–E).

To block radial glial division, we deleted *orc3* using the *nestin-cre* we employed above (Figure 1G–H″). Nestin is known to be expressed in neural as well as vascular cells, and several lines of *nestin-cre* transgenic mice, using the neural-specific *nestin* enhancer element, have been generated, with distinct expression patterns. To avoid *cre*-induced recombination in vascular cells, we selected the specific *nestin-cre* line used in our study for several reasons. First, several groups have independently confirmed that the *nestin-cre* line used in the present study does not induce recombination in vascular cells of the forebrain [17],[19],[29]. We have also directly assessed *cre* activity in the cortex and found that, despite near complete recombination in neural cells, no vascular recombination was observed (Figure S2). Furthermore, we observed increased EC proliferation in *orc3/nestin-cre* mutant brains (see results below), a phenotype opposite to what would have been predicted, had *orc3* been deleted in ECs. Lastly, we found that pericyte density along the cortical vasculature is quantitatively normal in *orc3* mutants (see results below), which indicates that *orc3* is not being deleted by *nestin-cre* in pericytes. Thus, these lines of evidence indicate that this *nestin-cre* is suitable for neural-specific deletion of *orc3*.

**Figure 2:**
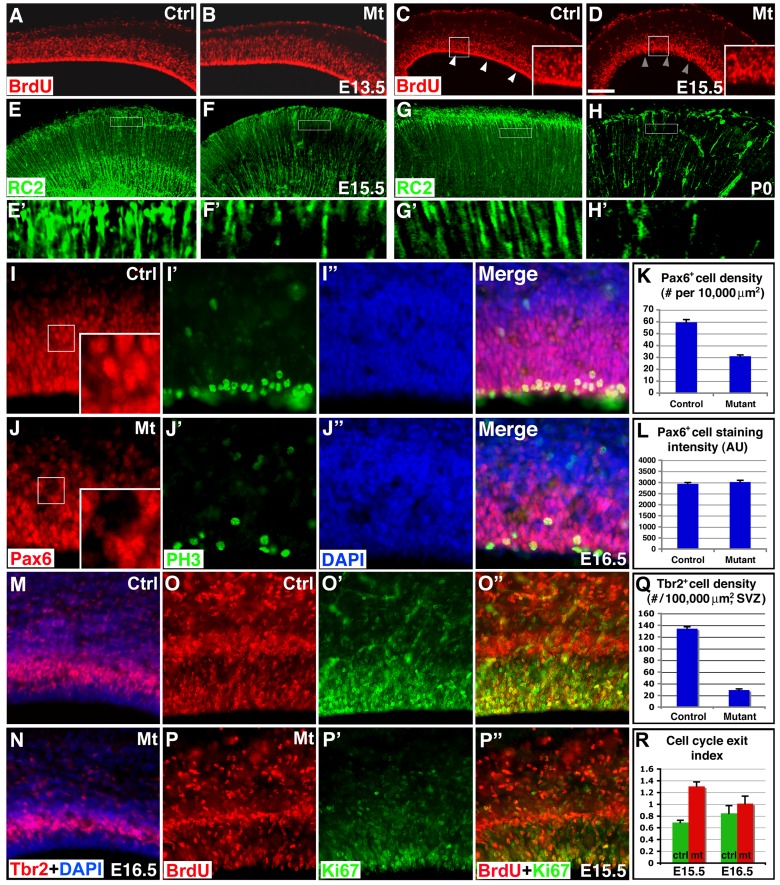
Blockade of cell cycle progression reduces cortical neural progenitors without affecting cell fate. (A–D) Effects of *orc3* deletion on radial glial division. BrdU labeling (in red) revealed no obvious defects in ventricular zone division at E13.5, but substantial reductions at E15.5, especially at the ventricular surface (arrowheads in C and D). (E–H) Effects of *orc3* deletion on radial glial density. RC2 staining (in green) revealed substantial reductions in radial glial density at E15.5 and near complete loss at P0. Boxed areas in (E–H) are shown in (E′–H′). Also notice more severe loss of radial glia in the mutant medial cortex (right side in H). (I–J″) Effects of *orc3* deletion on radial glial fate. Although reduced in number, mutant radial glia express normal levels of Pax6 (in red) (I and J) at E16.5. PH3 (in green) staining also revealed a reduced number of mitotic cells at the ventricular surface (I′ and J′). (K–L) Quantification of Pax6^+^ cell density and expression level. Significant differences were observed in Pax6^+^ cell density (*p* = 0.0003, *n* = 4) but not Pax6 staining intensity (*p* = 0.32, *n* = 20). Note cells are less densely packed in mutants. (M–N) Effects of *orc3* deletion on intermediate progenitors. Tbr2 staining (red) revealed severe reduction in intermediate progenitors in mutants at E16.5. (O–P″) Cell cycle exit analysis at E15.5. BrdU was administered at E14.5 followed by staining for BrdU (red in O, P, O″, and P″) and Ki67 (green in O′, P′, O″, and P″) at E15.5. (Q) Quantification of Tbr2^+^ cells in the subventricular zone at E16.5. Significant reductions were observed in mutants at E16.5 (*p* = 3×10^−15^, *n* = 9). (R) Cell cycle exit indices at E15.5 and E16.5. Significant increases were observed in mutants at E15.5 (*p* = 0.0001, *n* = 5), but not at E16.5 (*p* = 0.39, *n* = 5). Scale bar (in D): 200 µm for (A–D), 50 for µm for (E–H), and 60 µm for (I–J″) and (M–P″).

To determine effects of *orc3* deletion on cortical neural progenitors, we first evaluated cell proliferation (the effects of *orc3* mutation on cortical neural development, as analyzed in the following experiments, are summarized in Table S1). We found that *orc3* deletion by *nestin-cre* did not obviously perturb ventricular zone cell division at E13.5 (Figure 2A,B), but substantially reduced division at E15.5 (Figure 2C,D). The intense band of BrdU^+^ cells normally observed at the ventricular surface in controls is almost absent in mutants at E15.5. Indeed, quantification showed that the density of BrdU^+^ cells in the mutant ventricular zone was not significantly affected at E13.5 (control, 37.33±2.33/10,000 µm^2^; mutant, 37.75±2.06/10,000 µm^2^; *p* = 0.90, *n* = 4) but was significantly reduced at E15.5 (control, 51.83±3.52/10,000 µm^2^; mutant, 28.00±0.86/10,000 µm^2^; *p* = 0.0008, *n* = 6). Similar reductions were also observed in phospho-histone 3 (PH3) staining at the ventricular surface at E16.5 (control, 23.80±2.24/10,000 µm^2^; mutant, 4.25±0.25/10,000 µm^2^; *p* = 0.0009, *n* = 5) (Figure 2I′,J′). In addition, quantification of nuclei in the ventricular zone showed an over 20% reduction at E15.5 (control, 125.4±2.6 per field; mutant, 99.9±3.1; *p* = 4×10^−7^, *n* = 18). Furthermore, Ki67 staining also revealed a 24% reduction at E15.5 (control, 228.5±15.8 per field; mutant, 174±8.3; *p* = 0.02, *n* = 6; see also Figure 2O′,P′). *nestin-cre* induces fairly widespread recombination in the cortex at E12.5 [29]. Yet we did not observe substantial reductions in radial glial division until E15.5. This suggests that the delay may result from perdurance of *orc3* mRNA and/or protein. On the other hand, since by E15.5, the early stages of cortical angiogenesis are close to complete (Figure 1A–F), this provides a unique opportunity to determine the effects of radial glial loss on the late step of vessel stabilization.

To further evaluate effects of *orc3* deletion on radial glial development, we next assessed radial glial density. We found that radial glial density is substantially reduced, by ∼36% at E15.5 (control, 6.0±0.4/100 µm; mutant, 3.8±0.3/100 µm; *p*<0.005, *n* = 5) (Figure 2E–F′) and ∼58% at E16.5 (*p*<0.01, *n* = 3). By P0, radial glial fibers are only observed sporadically across the mutant cortex (Figure 2G–H′). Nonetheless, the overall organization appears intact, with radial glial endfeet properly anchored at the pia and parallel fibers spanning the cortical wall. Throughout these stages, the density of radial glia also appears consistently more severely affected in the medial than the lateral cortex (unpublished data, see also Figure 2H), suggesting earlier and/or more complete recombination by *nestin-cre* in the medial cortex. On the other hand, we did not observe obvious apoptosis (unpublished data), consistent with previous data showing that blockade of cell cycle progression in radial glia does not trigger significant cell death [33].

To determine whether cell cycle blockade affects radial glial progenitor fate, we next stained sections with antibodies against Pax6, a transcription factor regulating radial glial development [34]. We found that, although the number of Pax6^+^ cells is reduced in mutants (control, 59.5±2.4/10,000 µm^2^; mutant, 30.9±1.2/10,000 µm^2^; *p* = 0.004, *n* = 4), the expression level in individual cells remains normal at E16.5 (Figure 2I,J; quantified in Figure 2K,L). This is also supported by our Western blotting results showing that, although the level of total Pax6 protein is reduced in the mutant cortex, after normalizing against the number of radial glial progenitors, Pax6 protein level is comparable between controls and mutants (Figure S3). Staining intensity in individual radial glial cells for Nestin and Vimentin, intermediate filament proteins specific for radial glia, also appears unchanged (unpublished data). Furthermore, although the number of intermediate progenitors is reduced at E16.5 (control, 134.0±3.1/100,000 µm^2^; mutant, 29.1±1.9/100,000 µm^2^; *p* = 3×10^−15^, *n* = 9), their Tbr2 expression level appears unaffected in mutants (Figure 2M,N,Q). In addition, at E15.5, the number of Tbr2 positive intermediate progenitors is also quantitatively unaffected (control, 91.9±9.3/100,000 µm^2^; mutant, 80.7±10.3/100,000 µm^2^; *p* = 0.44, *n* = 7) (Figure S4Q,R,W). The normal expression of these dorsal forebrain markers also indicates that dorsoventral patterning is unaffected. This is further supported by our finding that expression of the ventral marker Dlx1 is also unaffected (Figure S4O,P). Lastly, the fates of neuronal progeny produced by radial glia also appear unperturbed (see results below). Thus, these results together indicate that blockade of neural cell division by *orc3* deletion substantially reduces radial glial number, but does not affect the specification and maintenance of their cell fate.

### Effects of Radial Glial Ablation on Cortical Neurogenesis

Radial glia are not only progenitors for nearly all cortical neurons and glia, but they are also scaffolds for neuronal migration [12],[13]. Thus, ablation of radial glia is likely to affect cortical neuron production as well as migration. Indeed, we observed significant reduction in cortical thickness in mutants at P12 (Figure S4A,B), which suggests potential defects in neuronal production. Analysis by BrdU birthdating revealed that the generation as well as migration of upper but not deeper layer neurons are substantially compromised (Figure S4C–H), consistent with the observed loss of radial glia after E15.5 (Figure 2). In addition, in accordance with unperturbed radial glial progenitor fate (Figure 2), we found that, although the number of upper layer neurons is reduced, the intensity in each cell of expression for Cux1, a transcription factor specific for layer II–V neurons, appears normal at P0 (Figure S4I,J). Expression in deep layer neurons of Ctip2, a marker for layer V–VI neurons, also appears unaffected at P0 (Figure S4K,L). Furthermore, in the embryonic cortex, although we observed increased cell cycle exit by mutant progenitors at E15.5 (Figure 2O–P″,R), the total number of cortical neurons is quantitatively normal at E16.5 (Cux1: control, 81.8±5.7 per field; mutant, 81.0±4.9; *p* = 0.70, *n* = 6; Ctip2: control, 279±16 per field; mutant, 276±15; *p* = 0.42, *n* = 6) (Figure S4S–V,X). This apparent lack of effects is likely due to the smaller number of progenitors present in mutants after E15.5, which may offset effects of increased cell cycle exit during this period. Lastly, at E16.5, we observed a normal pattern of layer-specific marker expression (Figure S4S–V). Thus, these results indicate that, although radial glial ablation results in compromised upper layer neuron production and migration, it largely spares neuronal fate specification.

### Ablation of Radial Glial Progenitors Results in Cortical Vessel Regression

**Figure 3 pbio-1001469-g003:**
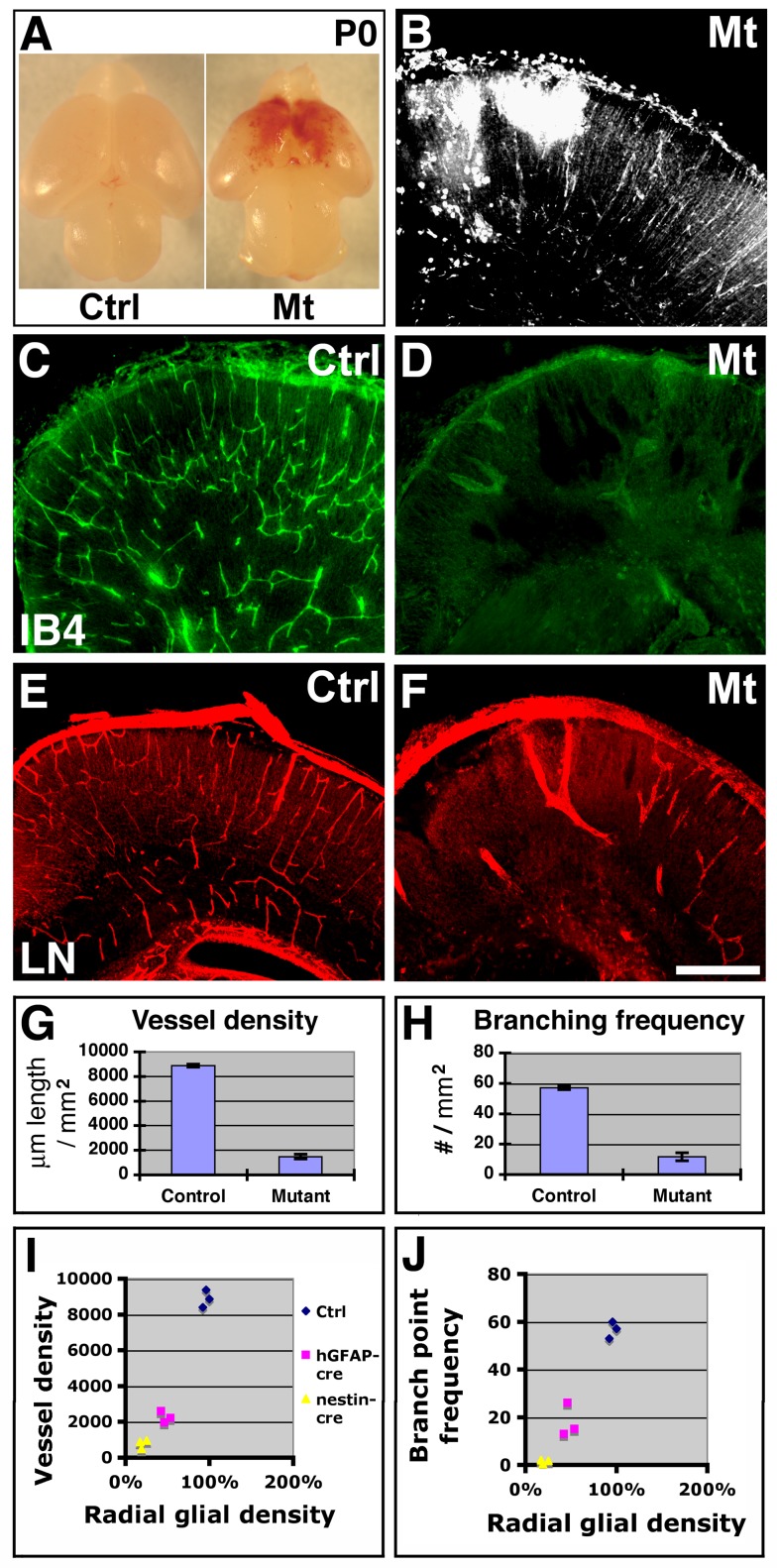
Ablation of neural progenitors results in defective cortical angiogenesis. (A–B) Neonatal brain hemorrhage in *orc3* mutants. Immunoglobulin reactivity further confirmed patterns of hemorrhage (B). (C–F) Vessel morphology at P0 along the anterior-posterior axis. IB4 (in green) and laminin (LN, in red) staining revealed severe loss of vessels in mutants in both the anterior (C and D) and posterior (E and F) cortex. (G–H) Quantitative analysis of cortical plate vessel density and branching frequency. Dramatic reductions in both vessel density (G) and branch point frequency (H) were observed (*p* = 1.76×10^−14^ and *p* = 1.01×10^−8^, respectively; *n* = 9). (I–J) Correlation between radial glial density and vessel density (I) as well as between radial glial density and vessel branching frequency (J) in the medial cortex of control, *orc3/nestin-cre*, and *orc3/hGFAP-cre* mutant neonates. The correlation coefficient was 0.97 for the former (I) and 0.98 for the latter (J). Scale bar (in F): 500 µm for (B–F).

To determine effects of radial glial ablation on cortical angiogenesis, we next compared vessel development in control and mutant brains. We observed neonatal cerebral hemorrhage in all mutants, a phenotype most severe in areas close to the midline (Figure 3A,B). This is consistent with our earlier observation of more severe radial glial loss in the medial cortex (see also Figure 2H). IB4 (Figure 3C,D) and laminin (Figure 3E,F) staining further revealed dramatic defects in cortical vascular network along the entire anterior-posterior axis (Figure 3D,F). Quantification showed that, at P0, vessel density across the cortex is reduced by ∼83% and branch point frequency by ∼79% in mutants (*p* = 1.76×10^−14^ and 1.01×10^−8^, *n* = 9) (Figure 3G,H). Similar to hemorrhage, vessel development also appears most severely affected in areas close to the midline (see Figure 3F). In addition, we sometimes observed tissue cavitations at P0. However, since we did not observe them at the embryonic stages (unpublished data), we assumed that these are due to secondary effects of vascular mal-development. Furthermore, we did not observe similar vascular defects in other regions of the CNS, including the hindbrain and the spinal cord (Figure S5). This is likely due to the late onset of expression of the *nestin-cre* relative to the (shorter) period of radial glial proliferation in these regions [35],[36]. Consistent with this interpretation, we found that, in contrast to the cortex (Figure 2E–F′), there were no significant changes in radial glial density in the hindbrain at E15.5 (control, 164.3±9.3/mm; mutant, 160.5±9.5/mm; *p* = 0.79, *n* = 4). To further assess the specificity of *orc3* deletion in the forebrain, we employed *hGFAP-cre*, a well-established neural-specific *cre*, the activity of which peaks around E14.5 in the neocortex, about 2 d after *nestin-cre* [37],[38]. We found that *orc3* deletion by *hGFAP-cre* results in a similar, albeit less severe, phenotype in cortical angiogenesis (Figure S6A–D). At P0, the vessel density and branching frequency are reduced by ∼71% and 67%, respectively, in the medial cortex (*p* = 5.25×10^−8^ and 8.48×10^−6^, *n* = 6), while no significant effects were observed in the lateral cortex (Figure S6A–D). This is consistent with another gradient of radial glial loss in *orc3/hGFAP-cre* mutant cortices (unpublished data), similar to, although shallower than, that observed in *orc3/nestin-cre* cortices. In addition, we also consistently observed significant, although also milder, hemorrhage near the midline of *orc3/hGFAP-cre* mutant cortices (unpublished data). Thus, these results altogether strongly indicate that neural cells play an essential role in cortical angiogenesis during late embryogenesis.

Since *nestin-cre* targets radial glia and, as a consequence, their progeny including intermediate progenitors, neurons, and astrocytes, this raises the question of whether defects in neuronal production and migration may be responsible for the observed vascular defects. To address this possibility, we examined neonatal brains from single and double mutants of *β1 integrin/emx1-cre* and *brca1/emx1-cre*, mutations that have been shown to result in disrupted cortical lamination and reduced neuron number, respectively [29],[39],[40]. We found that neither single nor double mutations in *β1 integrin* and *brca1* consistently affect cortical vessel density or branching frequency (Figure S6E–J). This indicates that defective angiogenesis following radial glial ablation is unlikely a result of reduced cortical neuronal number or defective migration, alone or in combination. This interpretation is also supported by previous analyses showing that, despite lamination defects, *reelin* mutation does not affect development of the cortical vasculature [41],[42]. Furthermore, specific loss of upper layer neurons in Pax6 mutants, similar to that in *orc3* mutants, also does not lead to obvious hemorrhage [43]. In addition, we found that deletion of *orc3* from intermediate progenitors and postmitotic neurons in the cortex, using *nex-cre* [44],[45], also has no obvious effects on vessel development (unpublished data). Lastly, at E16.5, when vessel regression begins, we found no quantitative defects in the density of either upper or deep layer cortical plate neurons (Figure S4S–V,X). Thus, these lines of evidence together strongly argue against the involvement of neuronal defects in *orc3* mutant vascular phenotype and suggest a role played by cortical neural progenitors.

Of the various cortical neural progenitors, studies showed that severe loss of intermediate progenitors does not result in obvious brain hemorrhage [46],[47]. This suggests that loss of radial glia is likely responsible for cortical angiogenesis defects in *orc3* mutants. To further evaluate this interpretation, we analyzed the quantitative relationship between radial glial density and vessel development in the medial cortex of controls as well as *orc3/nestin-cre* and *orc3/hGFAP-cre* mutants (Figure 3I–J). We found that, at P0, *orc3* deletion by *nestin-cre* results in a severe reduction in radial glial density in the medial cortex (∼79%), leading to a severe reduction in both vessel density (∼91%) and branch point frequency (∼98%). By contrast, *orc3* deletion by *hGFAP-cre* results in an intermediate reduction in radial glial density (∼51%), in correspondence to intermediate reductions in vessel density (∼75%) and branching frequency (∼68%). This indicates a positive relationship between radial glial density and cortical vessel density as well as branch point frequency. Indeed, further quantitative analysis showed that the correlation coefficient between radial glial density and vessel density (*r* = 0.97, *p* = 0.000015) as well as that between radial glial density and vessel branching frequency (*r* = 0.98, *p* = 0.000004) are both close to 1 (Figure 3I–J). Thus, these results demonstrate a tight, positive correlation of radial glial density with both cortical vessel density and branch point frequency. This not only further implicates a specific role of radial glia in cortical vessel development but also suggests the involvement of local cell-cell interaction.

**Figure 4:**
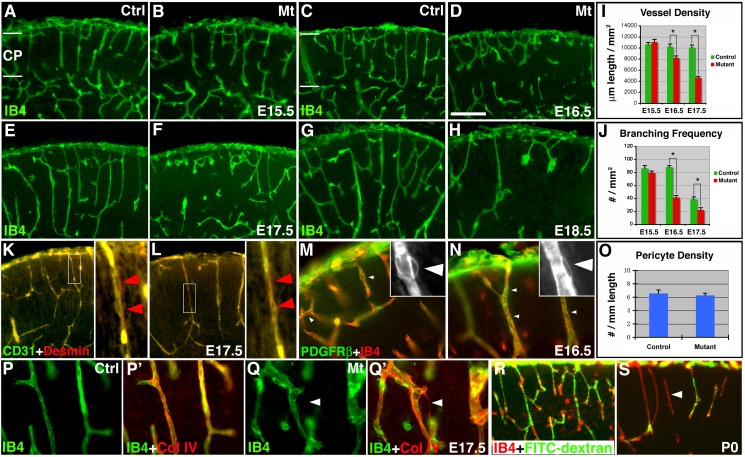
Ablation of neural progenitors results in cortical vessel regression independent of defects in pericyte recruitment. (A–H) Vessel morphology from E15.5 to E18.5. IB4 staining (in green) revealed no obvious defects at E15.5 (A and B) but significant defects at E16.5 (C and D), E17.5 (E and F), and E18.5 (G and H). (I–J) Quantification of vessel density and branching frequency from E15.5–17.5. Significant decreases (*) in both parameters were observed at E16.5 (*p* = 0.03 and 0.0003, *n* = 3) and E17.5 (*p* = 0.7×10^−5^ and 0.007, *n* = 5), but not at E15.5 (*p* = 0.52 and 0.25, *n* = 4). (K–N) Pericyte investment of vessels. Desmin staining (in red) surrounds CD31^+^ (in green) vessels similarly in mutants (L), as in controls (K). Boxed areas in (K and L) are shown in two right panels. PDGFRβ^+^ (in green) cells (some highlighted by arrowheads) were also associated with IB4^+^ (in red) vessels similarly in mutants (N), as in controls (M). PDGFRβ staining in boxed areas are shown as insets. (O) Quantification of PDGFRβ^+^ pericyte density. Pericytes along all vessels were quantified. No significant differences were observed (*p* = 0.62, *n* = 4). (P–Q′) Collagen IV staining. Collagen IV^+^ (in red) empty sleeves (arrowheads in Q′) were frequently observed in mutants (Q–Q′), in contrast to controls (P–P′). (R–S) Vessel perfusion. Neonates were perfused trans-cardially using 1% FITC-dextran (in green) followed by IB4 (in red) staining. Many vessels in mutant brains (S) were not well perfused (arrowhead in S), in contrast to those in controls (R). Scale bar (in D): 200 µm for (A–H), (K–N), and (R–S), 80 µm for (P–Q′).

To determine how vascular defects in *orc3/nestin-cre* mutants arise, we next examined cortical vessel morphology during embryogenesis (Figure 4A–H). We found that, at E15.5, vessel development appears completely normal, with the emergence of a primitive vascular network close to complete in all mutants (Figure 4A,B). This indicates that early cortical angiogenesis is not affected by *nestin-cre*–mediated *orc3* deletion, an interpretation consistent with our finding that neither neural progenitor proliferation nor radial glial density is substantially affected until after E15.5 (Figure 2A–F′). By contrast, at E16.5, we observed vessel defects in the vast majority of mutants. While some mutants showed relatively moderate deficits, most showed severe defects (Figure 4C,D). By E17.5 and E18.5, all mutants showed severe defects in vessel development (Figure 4E–H). Quantification revealed that the vessel density and branching frequency first begin to decrease at E16.5 and continue to decline afterwards (Figure 4I,J). Most importantly, we found that, even after adjustment for cortical area expansion, mutant vessel density still decreases by ∼30% from E16.5 to E17.5 (*p* = 0.0009, *n* = 5) and by another 39% from E17.5 to P0 (*p* = 0.0007, *n* = 5). This indicates that the total vessel length is being severely reduced during each of these intervals. Loss of radial glia during late embryogenesis therefore results in a distinct phenotype of vessel regression.

Since during early corticogenesis, radial glia promote CNS vessel development by expressing high levels of Wnt7a and Wnt7b, factors essential for initial vessel ingression into the neural tube [19],[23], our results above indicate that radial glia play a different role during late embryogenesis, by regulating nascent vessel stabilization. Consistent with this interpretation, after E14.5, Wnt ligand expression, especially that of Wnt7b, substantially shifts to the cortical plate [48], the main area of vessel development during these later periods. This suggests that radial glia may no longer play a primary role in promoting cortical plate vessel growth during late embryogenesis, a role likely taken over by neurons. To further assess our interpretation of vessel regression, we examined expression of Collagen IV, a basement membrane component frequently left behind by regressing vessels [24]. We found that, while there are rarely Collagen IV^+^ basement membrane sleeves in controls, the density of empty sleeves, albeit still low, is significantly increased in mutants (control, 0.72±0.45/mm^2^; mutant, 7.90±0.66/mm^2^; *p* = 1.5×10^−5^; *n* = 6) (Figure 4P–Q′). Furthermore, mutant vessels also appeared less well-perfused (Figure 4R,S). Thus, these results reinforce the interpretation that radial glia regulate cortical vessel stabilization during late embryogenesis and their loss results in vessel regression.

Pericytes are critical support cells for vessel development. Previous studies show that absence of pericytes, although resulting in an altered, torturous vessel morphology, does not affect either vessel density or branch point frequency in the developing brain [49]. Since we observed severe reductions in vessel density and branch point frequency (Figure 3), this suggests that potential defects in pericyte loss are unlikely a primary cause for vessel regression in *orc3* mutants. Nonetheless, to further assess their potential contribution, we examined pericyte investment of cortical vasculature. We found that, at E17.5, despite clear signs of vessel regression, cells positive for Desmin, a pericyte marker, similarly surround all cortical endothelia in mutants as in controls (Figure 4K,L). Comparable results were also obtained at E16.5, at the onset of vessel regression, using antibodies against PDGFRβ, another well-known pericyte marker (Figure 4M,N). Indeed, quantification showed that the density of PDGFRβ^+^ cells along all cortical plate vessels at E16.5 is statistically identical between controls and mutants (Figure 4O), arguing against pericyte loss or defective recruitment due to *orc3* deletion by *nestin-cre*. In addition, we observed a similar vascular phenotype in mutants where *orc3* was deleted using *hGFAP-cre* (Figure S6A–D), a *cre* line with well-established neural-lineage specificity [37],[38]. This further argues against primary defects in pericytes. Thus, these results indicate that pericyte defects do not play a primary role in vessel regression following radial glial ablation.

### Radial Glial Ablation Results in Ectopic Activation of Wnt Pathway in ECs

The above results indicate that loss of radial glia during late embryogenesis results in destabilization and regression of nascent brain vessels. To determine the underlying molecular mechanisms, we evaluated potential involvement of several pathways. VEGF is a survival factor for ECs in a number of tissues [50]–[53]. Thus, compromised VEGF expression may result in vessel regression. However, we found that the overall mRNA and protein levels of the three VEGF A isoforms are either not obviously changed at E16.5 (Figure S7) or even slightly increased at E17.5 (unpublished data), suggesting that the phenotype is unlikely a result from primary defects in the VEGF pathway. Similarly, angiopoietin 1 mRNA expression at E16.5 was also unchanged (Figure S7), arguing against primary defects in angiopoietin 1 signaling. A third candidate pathway is TGFβ signaling [54]. Indeed, perturbations of TGFβ signaling result in hemorrhage in a large number of mutants. However, no obvious vessel regression has been observed, including in the developing brain [17],[55],[56]. In *orc3* mutants, we observed severe vessel loss in the perinatal brain (Figure 3). Yet despite extensive hemorrhage, no quantitative reductions in total vessel length were observed in the perinatal brain following Smad4 deletion from the vasculature [56]. Furthermore, in Smad4 mutants, brain vessels appear obviously dilated throughout the capillary network [56]. Yet no similar dilations were observed in *orc3* mutants (see also Figure 4A–H). Thus, although defects in TGFβ signaling, if any, may contribute to the vascular phenotype, they cannot be solely or primarily responsible for vessel regression. These findings therefore suggest primary involvement of pathways other than VEGF, angiopoietin, or TGFβ signaling.

Canonical Wnt signaling has been implicated in several steps of vessel development, including initial neural-tube vessel ingression, retinal vessel stabilization, intersomitic vessel remodeling, and hyaloid vessel regression [19],[21]–[25]. In the embryonic neocortex, similar to the hindbrain [57], we found strong EC expression of a BAT-lacZ Wnt reporter during early development (Figure S8A–E) [58]. This suggests a role of Wnt signaling in early cortical angiogenesis. The expression, however, is strongly down-regulated after E16.5, coincident with onset of the later periods of vessel stabilization (Figure 1). In contrast to reductions in Wnt pathway activity, however, the expression of Wnt ligands, including Wnt7a and Wnt7b, continues in the cortex, even though substantially part of it, especially that of Wnt7b, shifts to the cortical plate [48]. Thus, these findings suggest that down-regulation of EC Wnt signaling, independent of changes in Wnt ligand expression levels, may play a role in cortical vessel stabilization during late embryogenesis.

**Figure 5:**
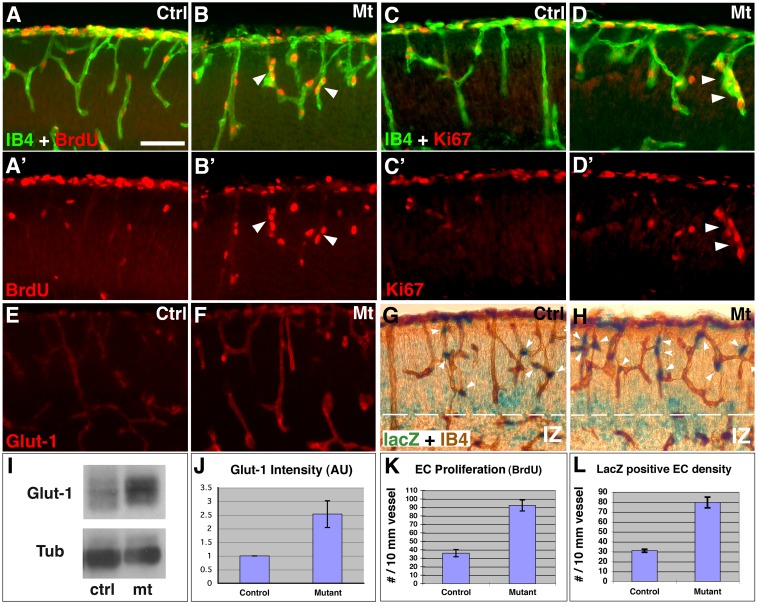
Increased proliferation and up-regulated canonical Wnt pathway activity in ECs following neural progenitor ablation. (A–B′) Cortical EC proliferation at E16.5. BrdU labeling (in red) revealed increased clusters (arrowheads) of BrdU^+^ ECs (IB4 in green) in mutants (B and B′). (C–D′) Ki67 staining at E16.5. Increased clusters of Ki67^+^ ECs (in red, arrowheads) were observed in mutants (D and D′). (E–F) Glut-1 expression in ECs at E16.5. Up-regulation of Glut-1 expression (in red) was observed in mutants (F). (G–H) Expression of Wnt reporter BAT-lacZ in ECs at E16.5. X-Gal reaction revealed increased numbers of lacZ^+^ (in blue, arrowheads) ECs (IB4 in brown) in mutants (H). BAT-lacZ up-regulation in neural cells appears largely restricted to the intermediate zone (IZ). (I–J) Western blot analysis of Glut-1 expression at E16.5. Stronger bands were observed in mutants (I). Quantification showed a >150% increase in mutants (J) (*p* = 0.03, *n* = 3). (K–L) Quantification of EC proliferation and BAT-lacZ expression at E16.5. The density of both BrdU^+^ (*p* = 0.001; *n* = 4) and lacZ^+^ (*p* = 0.001; *n* = 3) ECs is significantly increased in mutants. Scale bar (in D): 200 µm for (A–H).

Hyaloid vessel regression induced by Wnt signaling is associated with enhanced EC division [21],[22]. To determine whether this is the case in *orc3* mutants, we examined EC proliferation at E16.5 (Figure 5A–B′), the stage when vessel phenotypes first appear. In contrast to sporadic BrdU^+^ ECs in controls (Figure 5A,A′), we found frequent clusters of dividing ECs in mutants (Figure 5B,B′), which suggests increased EC proliferation. Indeed, quantification showed a >100% increase in the density of BrdU^+^ ECs (*p* = 0.001; *n* = 4) (Figure 5K). Similar increases were also observed using Ki67 antibodies (Figure 5C–D′). To more directly evaluate Wnt pathway activity, we examined expression of Glut-1, a blood brain barrier transporter regulated by Wnt signaling [19]. We found that Glut-1 staining is up-regulated by >65% in mutant vessels (*p* = 10^−16^, *n* = 22) (Figure 5E,F), suggesting elevation of Wnt signaling. This was further corroborated by Western blot analysis (Figure 5I,J). In addition, expression of LEF1, a canonical Wnt signal mediator as well as a transcriptional target expressed in spinal cord ECs [19], also appears up-regulated (unpublished data). Lastly, we employed the BAT-lacZ reporter for directly evaluating Wnt pathway activity in ECs (Figure 5G,H). We found that the density of lacZ^+^ ECs is approximately 150% higher in mutants than in controls (*p* = 0.001; *n* = 4) (Figure 5L). Furthermore, we consistently observed higher degrees of elevation of BAT-lacZ expression along mutant vessels in the medial cortex (unpublished data), corresponding to more severe angiogenesis defects in the midline region (Figure 3). Thus, these results demonstrate a close correlation between elevated EC Wnt signaling and vessel regression and strongly suggest a role of ectopic Wnt signaling in vessel regression.

To determine how EC Wnt pathway activity becomes elevated in *orc3* mutants, we next examined the expression of Wnt ligands and inhibitors in the cortex. We found that the levels of Wnt ligands Wnt7a and Wnt7b were not significantly changed at the mRNA level at E16.5 (Figure S8F), consistent with the finding that their expression has shifted substantially to cortical neurons at this stage [48] and that the number of cortical neurons is not significantly changed at E16.5 (Figure S4S–V,X). Interestingly, however, we found that the levels of several secreted Wnt inhibitors, including sfrp1, sfrp2, wif1, and Dkk1, were also not significantly affected at E16.5 (Figure S8F). Although this characterization was not exhaustive, this suggests that ectopic Wnt pathway activation in *orc3* mutants is not a result of an overall altered Wnt ligand/inhibitor ratio. This is also consistent with our finding that radial glial regulation of cortical vessel stabilization appears to depend on cell density (Figure 3I–J). In addition, although macrophage expression of Wnt7b has been found responsible for hyaloid vessel regression [21], we observed no obvious accumulation of microglia in the cortical plate until birth (Figure S9), days after the onset of vessel regression (Figure 4). This argues against a significant contribution of microglia. Thus, these results together suggest that radial glia likely regulate cortical vessel stabilization through local cell-cell interactions, which in turn inhibit Wnt signal reception by and/or transduction within ECs.

### Radial Glia Inhibit EC Wnt Signaling in a Contact and Stage-Dependent Manner

**Figure 6:**
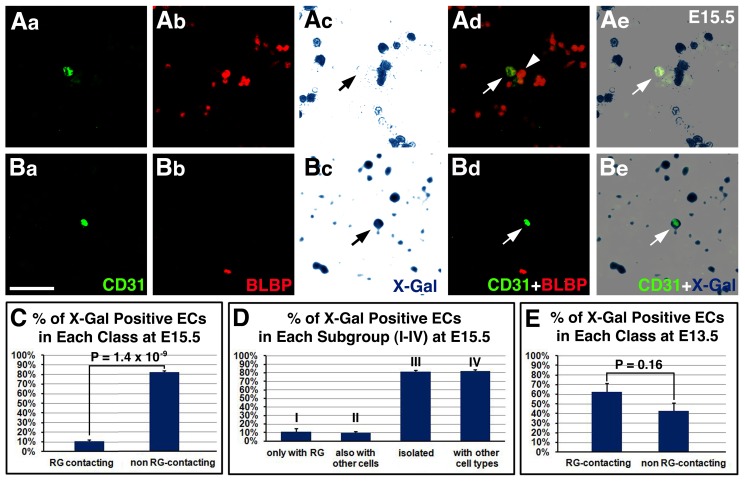
Contact-dependent suppression of EC Wnt signaling by radial glia at E15.5 but not at E13.5. (Aa–Be) Effects of contact with radial glia on EC Wnt pathway activity at E15.5. Cell cultures were stained for BAT-lacZ by X-gal reaction (in dark blue, in Ac, Ae, Bc, Be), followed by staining for EC marker CD31 (in green in Aa, Ad, Ba, Bd) and radial glial marker BLBP (in red in Ab, Ad, Bb, Bd). (Aa–Ae) Example of ECs (arrows) contacting radial glia (arrowhead) and showing minimal lacZ expression. (Ba–Be) Example of ECs (arrows) not contacting radial glia and showing strong lacZ expression. (C–D) Quantification of effects of radial glial interaction on EC BAT-lacZ expression at E15.5. The vast majorities of ECs interacting with radial glia (total 110 cells) do not express BAT-lacZ, while those that do not contact radial glia (total 133 cells) are overwhelmingly X-Gal^+^ (*p* = 1.4×10^−9^, *n* = 5) (C). Analysis of subgroups by ANOVA followed by Tukey's post hoc test also shows similarly significant effects of interaction with radial glia, but no significant effects of interactions with nonradial glia [*p*>0.05, between groups I (46 cells) and II (64 cells), as well as between groups III (61 cells) and IV (72 cells)] (D). (E) Quantification of effects of radial glial interaction on EC BAT-lacZ expression at E13.5. No significant differences were observed between the proportions of ECs contacting (total 76 cells) and not contacting radial glia (total 85 cells) that express BAT-lacZ (*p* = 0.16, *n* = 3). Scale bar (in Ba): 80 µm for (Aa–Be).

To more directly assess the nature of radial glial regulation of EC Wnt signaling, we next employed in vitro culture, using dissociated cortical cells from BAT-lacZ reporter mice. We first assessed effects of radial glia on EC Wnt signaling at E15.5. We evaluated Wnt pathway activity by X-gal reaction and identified ECs and radial glia by CD31 and BLBP staining, respectively (Figure 6Aa–Be). In these cultures, we observed strong X-gal staining in many cells, indicating robust Wnt pathway activation. To confirm effectiveness of cell dissociation especially of pericytes, we double-stained for CD31 and NG2. We found that less than 3% of ECs were associated with pericytes. This indicates that under these conditions, ECs and pericytes are near completely separated. Next, we evaluated effects of radial glial interaction on EC BAT-lacZ expression. We found that ECs in contact with radial glia overwhelmingly showed minimal BAT-lacZ activity (Figure 6Aa–f), while those not in contact with radial glia (Figure 6Ba–f) showed strong lacZ expression. Quantitative analysis confirmed a highly significant inhibitory effect of radial glial contact on EC Wnt pathway activity (Figure 6C,D). These results thus indicate that radial glia inhibit EC Wnt signaling in a contact-dependent manner at E15.5. Importantly, in these experiments, we found no significant effects on Wnt pathway activity by EC interaction with nonradial glial cells (Figure 6D), which at this stage consist mainly of cortical neurons. This further indicates the specificity of radial glial regulation of EC Wnt signaling. Interestingly, at E13.5, we found no similar inhibitory effects of radial glial contact (Figure 6E), which suggests a stage-specific interaction between radial glia and ECs. Thus, these results altogether demonstrate a direct and specific role of radial glia in inhibiting cortical EC Wnt signaling during late embryogenesis.

### Activation of Wnt Signaling Induces, While Attenuation Substantially Suppresses, Vessel Regression

To determine whether aberrant Wnt pathway activation is responsible for vessel regression in *orc3* mutants, we next sought to directly activate Wnt signaling. Glycogen synthase kinase 3β (GSK-3β) is a key component of canonical Wnt pathway, and inhibition of GSK-3β by LiCl mimics Wnt pathway activation in many developmental contexts [59],[60]. To assess effects of GSK-3β inhibition on Wnt signaling, we treated E16.5 embryos with LiCl. We found that LiCl quickly up-regulated expression of several Wnt target genes/reporters, a change most prominent in cortical ECs (Figure S10A–I). Indeed, we found that, about 10 h after treatment, the density of BAT-lacZ^+^ ECs is significantly increased, to ∼55% higher than in controls (Figure S10A–C). This increase, however, is not nearly as dramatic as that in *orc3* mutants (Figure 5L), consistent with the finding that LiCl is quickly cleared from the plasma in mice [61]. In addition, we found that, unlike in *orc3* mutants where EC BAT-lacZ expression is most dramatically up-regulated in the medial cortex, the up-regulation by LiCl appears more uniform (unpublished data), consistent with the fact that LiCl is being delivered by the brain vasculature. Furthermore, we found that the level of Glut-1 expression is also significantly elevated by LiCl (Figure S10D–F). Lastly, we found that LiCl also significantly up-regulated EC proliferation (Figure S10G–I). Thus, these results indicate that LiCl treatment efficiently elevates EC Wnt signaling in the embryonic cortex. To assess the specificity of LiCl treatment, we next examined its effects on the expression of a panel of Wnt ligands and extracellular inhibitors. We found that LiCl did not significantly affect the expression of either the Wnt ligands Wnt7a and Wnt7b or the inhibitors sfrp1, sfrp2, wif1, and Dkk1, as assayed by qRT-PCR (Figure S11A). Furthermore, we found that LiCl treatment also had no significant effects on either the expression pattern or the density of Cux1 or Ctip2 positive neurons (Figure S11B–E,H–I). Thus, these results together indicate that LiCl provides a relatively specific and efficient tool for activating EC Wnt signaling throughout the embryonic cortex.

**Figure 7:**
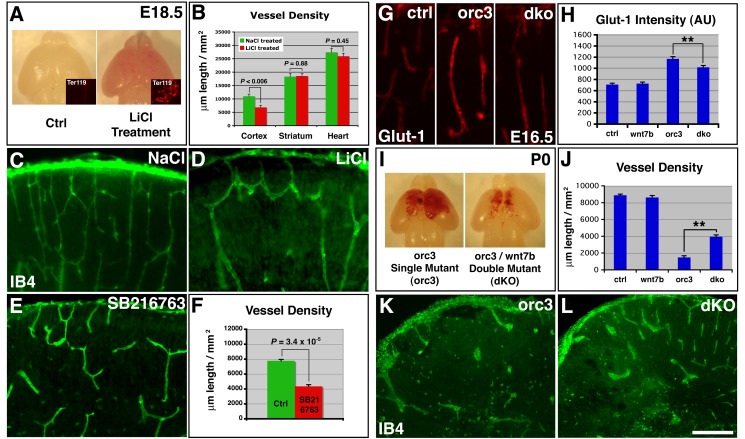
Activation of canonical Wnt signaling induces, while attenuation substantially suppresses, vessel regression. (A–D) Effects of LiCl-mediated Wnt pathway activation on vessel development. LiCl treatment at E16.5–17.5 induces hemorrhage throughout E18.5 brains (A). Ter119 staining confirmed microhemorrhage throughout LiCl-treated brains (insets in A). Quantification showed significant decreases in vessel density in the cortex (*p* = 0.0057, *n* = 7), but not in the striatum (*p* = 0.88, *n* = 6) or the heart (*p* = 0.45, *n* = 3) following LiCl treatment (B). Vessel morphology in NaCl- and LiCl-treated E18.5 cortices is shown in (C and D). (E–F) Effects of SB216763-mediated Wnt pathway activation on vessel development. SB216763 treatment at E15.5–17.5 induces vessel loss in E18.5 brains (E). Quantification showed significant decreases in vessel density in the cortex (*p* = 3.4×10^−5^, *n* = 4) (F). (G–H) Effects of *wnt7b* mutation on Glut-1 expression in *orc3* mutants. Introduction of *wnt7b* into *orc3* mutant background suppresses increases in Glut-1 expression at E16.5 (G). Quantification of Glut-1 expression and analysis by ANOVA followed by Tukey's post hoc test shows that *wnt7b* mutation alone has no significant effects but suppresses Glut-1 expression in *orc3* mutants (** *p*<0.01, *n* = 13) (H). (I–L) Effects of *wnt7b* mutation on vessel regression in *orc3* mutants at P0. Introduction of *wnt7b* into *orc3* mutant background suppresses brain hemorrhage (I). Quantification of vessel density and analysis by ANOVA followed by Tukey's post hoc test shows that *wnt7b* mutation alone has no significant effects on vessel density but suppresses vessel regression in *orc3* mutants (**p<0.01, *n* = 4) (J). Cortical vessel morphology in *orc3* single and *orc3/wnt7b* double mutants is shown in (K and L). Scale bar (in J): 200 µm for (C–E), 100 µm for (G), and 500 µm for (K–L).

To determine effects of GSK-3β inhibition on vessel stabilization, we next examined vessel density and branching frequency at E18.5, following LiCl treatment starting at E16.5, in comparison to NaCl treatment (Figure 7A–D). We found that LiCl treatment results in mild hemorrhage throughout the cortex, which, as a consequence, appears pink in color, a phenotype that we confirmed by Ter119 staining (Figure 7A). These moderate effects are consistent with the mild elevation of Wnt signaling by LiCl (Figure S10A–C). The widespread nature of the hemorrhage also appears consistent with activation of EC Wnt signaling by LiCl throughout the cortex. These results thus indicate that ectopic activation of EC Wnt signaling can result in hemorrhage. To determine effects on vessel stabilization, we next quantified cortical vessel density at E18.5 in control and treated embryos. We found that LiCl treatment results in a vessel density not only lower than that of E18.5 controls (Figure 7B) but also lower than that of E17.5 controls (E17.5 control, 10,112±440 µm/mm^2^; E18.5 LiCl treated, 6,832±794 µm/mm^2^; *p* = 0.004, *n* = 7). This indicates that elevation of EC Wnt signaling by LiCl can also result in vessel regression. To assess the specificity, we examined effects of LiCl on vessel development outside the cortex. We found that, despite its dramatic impact on the cortex, LiCl had no significant effects on vessel density in either the striatum or the heart at this stage (Figures 7B and S10J–O). To further address the issue of specificity, we also determined effects of SB216763, a more specific GSK-3β inhibitor. We found that, similar to LiCl, SB216763 also had no significant effects on either the expression pattern or the density of Cux1 or Ctip2 positive neurons in the cortex (Figure S11F–I). However, SB216763 treatment resulted in significant reductions in vessel density (control, 7,772±199 µm/mm^2^; SB216763 treated, 4,351±229 µm/mm^2^; *p* = 3.4×10^−5^, *n* = 4) as well as branching frequency (control, 37.8±2.3/mm^2^; SB216763 treated, 15.8±2.0/mm^2^; *p* = 0.0004, *n* = 4) in E18.5 cortices (Figure 7E–F). Thus, these results altogether indicate that Wnt pathway activation is sufficient to trigger cortical vessel regression, suggesting that ectopic Wnt pathway activation likely plays a significant role in vessel regression following radial glial ablation.

To determine whether ectopic Wnt pathway activation indeed contributes to vessel regression in *orc3* mutants, we next sought to attenuate Wnt signaling (Figure 7G–L). Wnt7a and Wnt7b are two redundant Wnt ligands expressed in the embryonic cortex. They are initially expressed in radial glial progenitors during early corticogenesis but shift expression to cortical plate neurons after E14.5 [19],[23],[48]. We reasoned that, even though Wnt7a and Wnt7b are functionally redundant during normal CNS angiogenesis, removing one of them in the *orc3* mutant background may still attenuate elevated Wnt signaling. To this end, we introduced homozygous *wnt7b* mutation into the *orc3* mutant background. We found that, although deleting *wnt7b* alone has no significant effects on cortical vessel Glut-1 expression, deleting *wnt7b* from *orc3* mutants significantly suppresses the up-regulation of Glut-1 expression (*p*<0.01, *n* = 13; ANOVA and Tukey's post hoc test) (Figure 7G,H). This indicates that *wnt7b* mutation suppresses the up-regulation of EC Wnt signaling in *orc3* mutants. On the other hand, we observed no significant effects of *wnt7b* deletion on the number of Tbr2^+^ cells in either the wild-type or *orc3* mutant background (*p*>0.1, *n* = 10), which suggests a relatively specific effect on ECs. Consistent with the suppression effects of *wnt7b* deletion on EC Wnt signaling, we also found that, although deleting *wnt7b* alone has no significant effects on cortical vessel development (Figure 7J), deleting *wnt7b* from *orc3* mutants not only substantially ameliorates cerebral hemorrhage (hemorrhage area in cross-sections: *orc3* mutant, 0.146±0.021 mm^2^; *orc3/wnt7b* dKO, 0.059±0.006 mm^2^; *p* = 0.003, *n* = 5) (Figure 7I), but also substantially restores cortical vessel density (*orc3* mutant, 1,487±189 µm/mm^2^; *orc3/wnt7b* dKO, 3,945±218 µm/mm^2^; *p*<0.01, *n* = 4), as well as branch point frequency (*orc3* mutant, 11.8±2.7/mm^2^; *orc3/wnt7b* dKO, 24.8±2.6/mm^2^; *p*<0.01, *n* = 4) (Figure 7J–L). The suppression, however, is not complete, suggesting that, even in the *orc3* mutant background, there are still substantial degrees of redundancy between Wnt ligands. Thus, these results indicate that ectopic activation of EC Wnt signaling plays a large role in vessel regression following radial glial ablation. Radial glia therefore appear to stabilize nascent cortical plate vessels in large part through inhibiting EC Wnt signaling.

### Ectopic Wnt Pathway Activation May Affect Vessel Regression in Part Through Matrix Metalloproteinase 2 (MMP-2)

**Figure 8:**
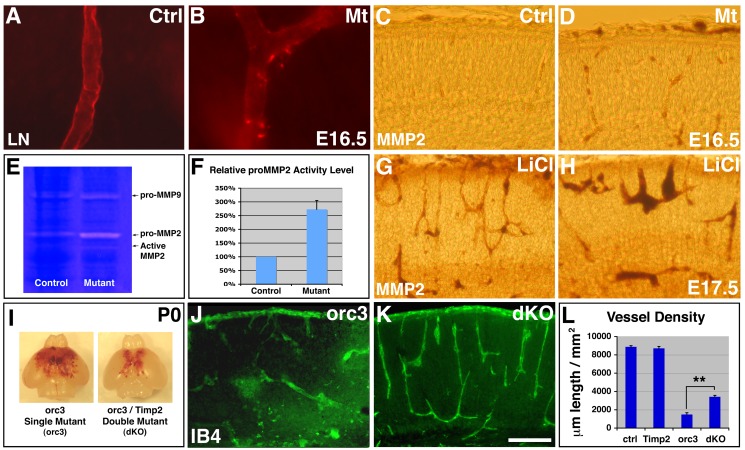
Ectopic Wnt signaling may destabilize cortical vessels in part through up-regulation of MMP-2. (A–B) Effects of radial glial ablation on vessel basement membrane. Staining with highly diluted anti-laminin (LN) antibodies (in red) revealed even labeling along control vessels (A), but frequent bright puncta in mutants (B). (C–D) MMP-2 expression along vessels at E16.5. Antibody staining (in brown) revealed low levels of MMP-2 along control vessels (C), which appear elevated in mutants (D). (E–F) Gelatin zymography. Full-length pro-MMP-2 activity appears substantially up-regulated in mutants at E16.5. Cleaved MMP-2 was also detected in mutants but not in controls. Quantification confirmed an over 170% increase in pro-MMP-2 levels (*p* = 0.006; *n* = 3) (F). (G–H) MMP-2 expression in brains treated with LiCl at E17.5. LiCl dramatically elevated MMP-2 expression along cortical vessels. (I–L) Effects of *Timp2* mutation on vessel regression in *orc3* mutants. Introduction of *Timp2* mutation into *orc3* mutant background substantially suppressed brain hemorrhage (I) and restored cortical vessel network (K and J). Analysis by ANOVA followed by Tukey's post hoc test shows that *Timp2* mutation alone has no significant effects on vessel density, but suppresses vessel regression in *orc3* mutants (** *p*<0.01, *n* = 6) (L). Scale bar (in K): 70 µm for (A–B) and 200 µm for (C–D, G–H, and J–K).

Canonical Wnt signaling directly promotes, at the transcriptional level, expression of several matrix metalloproteinases (MMPs), including MMP-2/9, membrane type 1 MMP (MT1-MMP), and MT3-MMP [62]. Since precise regulation of MMP activity is crucial for the delicate balance between vessel basement membrane breakdown and stabilization during angiogenesis [63], elevated MMP expression may result in excessive basement membrane breakdown, leading or contributing to vessel regression. Indeed, along *orc3* mutant vessels, we frequently observed bright puncta of laminin staining (Figure 8A,B) (0.56±0.30/mm vessel length for controls; 2.99±0.29/mm for mutants; *p* = 0.006, *n* = 10), which suggests excessive basement membrane breakdown.

To assess the role of MMPs, we first examined MMP-2 expression in cortical plate vessels during normal development. Consistent with moderate levels of Wnt pathway activity in wild-type ECs at E15.5 and E16.5 (see Figure 5G), we found low numbers of vascular cells expressing MMP-2 at these stages (Figure 8C). By contrast, with elevated EC Wnt pathway activity in *orc3* mutants (see Figure 5H), we found substantially increased numbers of vascular cells expressing MMP-2 (Figure 8D). To corroborate these observations, we performed gel zymography (Figure 8E,F). We found that the activities of both full-length pro-MMP-2 and cleaved MMP-2 are dramatically elevated in mutants at E16.5, while the activity of pro-MMP-9, another MMP expressed in the cortex, appears moderately increased (Figure 8E). Quantification showed an over 170% increase in pro-MMP-2 activity (*p* = 0.006; *n* = 3) (Figure 8F), as well as a roughly 90% increase in pro-MMP-9 activity (*p* = 0.007; *n* = 3). This suggests that elevated MMP-2/9 activity may both play a role in vessel regression. Furthermore, we found that, in brains treated with LiCl, MMP-2 expression is also dramatically up-regulated (Figure 8G,H). On the other hand, unlike MMP-2 and MMP-9, MT1-MMP expression appears not significantly changed (Figure S12A). Thus, these results indicate that, during corticogenesis, Wnt signaling positively regulates MMP-2 (and possibly MMP-9) expression in vessels, while radial glia suppress MMP-2/9 expression.

To assess the functional significance of MMP-2 up-regulation, we sought to block MMP-2 activity in *orc3* mutants. Timp2, a tissue inhibitor of MMPs, is essential for the cleavage and activation of pro-MMP-2 [64],[65]. We confirmed that, in *Timp2* mutants, cleaved MMP-2 is completely absent (Figure S12B). To block MMP-2 activation, we introduced homozygous *Timp2* mutation into *orc3* mutant background and evaluated vessel development (Figure 8I–L). We found that *Timp2* mutation alone does not significantly affect vessel development (Figure 8L). However, introduction of homozygous *Timp2* mutation greatly reduces the extents of brain hemorrhage in comparison to that in *orc3* single mutants (Figure 8I). Quantification showed that the cross-section area covered by hemorrhage is significantly reduced in double mutants (*orc3* mutant, 0.146±0.021 mm^2^; *orc3/Timp2* dKO, 0.068±0.006 mm^2^; *p* = 0.007, *n* = 5). Introduction of *Timp2* mutation also significantly restores vessel morphology as well as density in the *orc3* mutant cortex (Figure 8J,K). Quantification showed that both vessel density (*orc3* mutant, 1,487±189 µm/mm^2^; *orc3/Timp2* dKO, 3,420±157 µm/mm^2^; *p*<0.01, *n* = 6; ANOVA and Tukey's post hoc test) and branch point frequency (*orc3* mutant, 11.8±2.7/mm^2^; *orc3/Timp2* dKO, 25.7±2.1/mm^2^; *p*<0.01, *n* = 6) are significantly higher in *orc3/Timp2* double mutants than in *orc3* single mutants (Figure 8L). The suppression, however, is partial, suggesting potential contribution by other Wnt target genes. Thus, these results argue that MMP-2 up-regulation may play a functionally significant role in vessel regression following radial glial ablation. They indicate that nascent cortical vessel stabilization by radial glia may be mediated, in part, by inhibition of MMP-2 expression in vascular cells.

## Discussion

Blood vessels in the brain and throughout the body show tissue-specific patterns of growth, branching, and differentiation, suggesting intimate regulation by target-specific cell types. In this article, we show that, during late embryogenesis, radial glial progenitors in the developing cortex participate in this process by regulating the stabilization of nascent brain vascular network, via inhibition of EC Wnt signaling. First, we show that vascular development in the embryonic cortex undergoes a phase of sprouting followed by stabilization, in a pattern coordinate with radial glial organization (Figure 1). We find that ablation of radial glia results in vessel regression in a density-dependent manner (Figures 2–4), concomitant with ectopic activation of Wnt signaling in ECs (Figure 5). We also find that radial glia inhibit EC Wnt signaling in a contact and stage-dependent manner (Figure 6). Direct activation of Wnt signaling, on the other hand, is sufficient to induce similar vessel regression, while attenuation of Wnt signaling significantly suppresses regression (Figure 7). Lastly, we find that vessel stabilization may be mediated, in part, through inhibition of endothelial MMP-2 expression (Figure 8). These results thus reveal a previously unrecognized role of radial glia in brain vessel development and provide novel insights into the molecular mechanisms through which brain-specific cell types regulate vessel stabilization, a crucial step of vascular development, patterning, and repair that remains poorly understood. They have also significant implications for a number of neurovascular and related diseases.

### Radial Glial Regulation of Cortical Vessel Stabilization

Previous studies have observed close interactions between radial glia and blood vessels during development [15],[16], suggesting a role of radial glia in EC guidance. Our results show that radial glia also play an unexpected role in cortical vessel stabilization, a later step of brain angiogenesis. First, we show that the *nestin-cre* we use specifically targets neural cells, while sparing the cortical endothelia (Figures S2), confirming work by others [19]. This indicates that vessel regression in *orc3/nestin-cre* mutants is due to primary defects in neural but not ECs. Second, we observe increased proliferation of cortical ECs in mutants, which further argues against *orc3* deletion in ECs (Figure 5). In addition, we observe normal pericyte density along vessels (Figure 4), which argues against *orc3* deletion by *nestin-cre* in pericytes. Furthermore, we find severely reduced cortical vessel density and branch point frequency in *orc3/nestin-cre* mutants (Figure 3), a phenotype not observed in mutants without pericytes [49]. Lastly, we find that deletion of *orc3* using an independent neural-specific *hGFAP-cre* also results in similar vascular defects (Figures 3I–J and S6). Thus, these lines of evidence strongly argue for a role of neural cell types in cortical vessel stabilization.

Among the cortical neural cell types, our results strongly argue against involvement of postmitotic neurons. By taking advantage of *β1 integrin* and *brca1* mutants, we show that general defects in cortical neuron production and migration, alone or in combination, do not result in vessel regression (Figure S6). This is consistent with previous results from analyzing *reelin* mutants [41],[42]. Furthermore, we find that specific deletion of *orc3* from cortical neurons also has no effects on vessel development. Lastly, at E16.5, when vessel regression begins, we found no quantitative defects in the density of either upper or deep layer cortical plate neurons (Figure S4S–V,X). These results thus strongly argue against involvement of cortical neurons and implicate a role by neural progenitors. Of the neural progenitors, previous studies show that severe loss of intermediate progenitors does not result in similar brain hemorrhage [46],[47]. We also did not observe obvious defects following *orc3* deletion by *nex-cre* from intermediate progenitors and neurons. This suggests a likely role by radial glia. Indeed, our quantitative analysis shows a strong, positive correlation between radial glial density and vessel density as well as branch point frequency (Figure 3I–J). Our cell culture experiments further demonstrate a stage-specific and contact-dependent inhibition by radial glia, but not by other cell types, of EC Wnt signaling (Figure 6), a pathway that we also functionally implicate in vessel regression (Figures 7 and 8). Thus, these results strongly indicate that radial glia are the neural cell type responsible for cortical vessel stabilization during late embryogenesis.

### Regulation of Vessel Development by Canonical Wnt Signaling

Canonical Wnt signaling has been implicated in several steps of vessel development, including CNS vessel ingression, blood brain barrier differentiation, retinal vessel stabilization, intersomitic vessel remodeling, and hyaloid vessel regression [19],[21]–[25]. Our results argue that Wnt signaling down-regulation in ECs is essential for cortical vessel stabilization during late embryogenesis. We observe a tight correlation between ectopic Wnt pathway activation and vessel regression. We find that EC expression of Wnt target genes/reporters is substantially elevated at the onset of vessel regression (Figure 5). We also find that radial glia normally inhibit cortical EC Wnt signaling during late embryogenesis (Figure 6). Furthermore, we find that direct activation of Wnt pathway induces vessel regression, while attenuation of Wnt signaling significantly suppresses regression (Figure 7). Lastly, ectopic activation of Wnt signaling up-regulates MMP-2 expression in ECs, while blocking MMP2 activity using *Timp2* mutation appears to substantially suppress vessel regression (Figure 8). Thus, these results strongly indicate that down-regulation of EC Wnt signaling may be a key step through which radial glia regulate cortical vessel stabilization.

The multiple and sometimes opposite roles played by canonical Wnt signaling during vascular development suggest that Wnt pathway activation may elicit, in a stage- and tissue-dependent manner, dramatically different responses in ECs. This raises the question of how these distinct effects are mediated in the same cell type. Our findings that Wnt signaling regulates MMP-2 expression may provide some clues. In the CNS, vessel ingression from outside the neural tube necessitates penetration of the neural tube basement membrane and thus likely increased expression of extracellular matrix degrading enzymes such as MMPs, the activities of which are known to facilitate angiogenesis [66]. By contrast, vessel stabilization following initial formation likely requires not only assembly but also maintenance of basement membrane and thus, as a prerequisite, tight control of MMP activity [63]. In this light, it would appear to make sense that activation of Wnt signaling is necessary for the initial vessel ingression into the neural tube as well as, potentially, their continued growth in the neural tissues [19],[23], while Wnt signaling down-regulation may be essential for vessel stabilization after their initial formation (our results).

### Vessel Stabilization in Development and Disease

Patterning of cellular networks during development typically involves guided initial growth as well as subsequent selective stabilization [3],[4]. As such, it is likely that tissue-specific patterning of vascular networks also involves similar mechanisms. Indeed, a large number of axon guidance cues have been implicated in recent years in directed vessel growth [5],. In contrast, little is known about what roles selective stabilization may play in vascular patterning and how it is regulated. In the neonatal retina, pericyte recruitment and astrocyte VEGF signaling regulate hyperoxia-induced vascular pruning, a process that matches local vessel density with oxygen supply level [53],[67]. In the developing eye, pericyte signaling is also responsible for macrophage-dependent hyaloid vessel regression [21],[22]. However, it remains unknown how tissue-specific cell types regulate vessel stabilization. Our results show that, in the developing cortex, radial glia are the tissue-specific cell type responsible for vessel stabilization. Our results also implicate contact-dependent radial glial down-regulation of EC Wnt signaling in this process. This therefore provides novel insights into not only the cellular but also the molecular mechanisms that coordinate tissue development and vascular patterning in the nervous system and potentially throughout the body.

Increasing evidence suggests that cerebrovascular dysfunction, including vessel regression, plays an important role in the pathogenesis of a significant number of developmental and degenerative diseases [68],[69]. For example, perinatal stroke, which includes hemorrhagic stroke, affects 1 in 1,600 to 5,000 live births and often results in long-term disabilities [69]. Yet little is known about the mechanisms underlying these conditions. Our results raise the possibility that dys-regulated neural progenitor and Wnt signaling may play a role. Consistent with this, recent studies show that cell-autonomous functions of CCM3 in neural cells play a significant role in the pathogenesis of cerebral cavernous malformation [20]. In aged humans, reduced capillary density, and even collapsed or degenerated endothelia, have been observed in Alzheimer's brains [70]. Missing vasculatures have also been detected, at sites of amyloid plaques, in models of Alzheimer's disease [71]. These findings highlight a role of vessel regression in the pathogenesis of Alzheimer's disease, while our results suggest that studies on the possible role of dys-regulated neural-to-vascular and Wnt signaling may be informative. Thus, further investigation into vessel stabilization by radial glia may not only enhance knowledge of mechanisms that coordinate neural and vascular development and function in the normal brain, but may also facilitate better understanding of human diseases.

## Materials and Methods

### Molecular Biology

BAC recombineering technology was employed for generating conditional *orc3* allele. qRT-PCR was performed according to the manufacturer's instructions (see Text S1 for details).

### Mouse Breeding and Pharmacology

This study was approved by the Animal Care and Use Committee of the University of Wisconsin–Madison (Animal Welfare Assurance #A3368-01). *nestin*-*cre* (#003771), *Timp2*, *wnt7b*, as well as *BAT-lacZ* and *mTomato/mEGFP* reporters were purchased from the Jackson Lab. *nestin-cre* was introduced paternally into the *orc3* background for phenotypic analyses. Males triply heterozygous for *orc3*, *nestin-cre*, and *Timp2* or *wnt7b* were crossed to females doubly heterozygous for *orc3* and *Timp2* or *wnt7b*, to produce double mutants. *emx1-cre* and *β1 integrin* conditional alleles were as published previously [39]. *brca1* conditional alleles were purchased from the National Cancer Institute Mouse Models of Human Cancers Consortium repository. For LiCl and NaCl injection, pregnant females were treated on E16.5 at 17 µmoles/g body weight (gbw), followed by three treatments on E17.5 (once every 5 h) at 10 µmoles/gbw, and embryos were collected on E18.5. For SB216763 injection, pregnant females were treated once on E15.5 at 0.27 µmole/gbw, followed by two treatments each on E16.5 and E17.5. BrdU was injected at 100 µg/gbw. Animal use was in accordance with institutional guidelines.

### Immunohistochemistry

Stainings were performed as described previously [39] and analyzed under a Nikon *eclipse* Ti microscope. For 3-D reconstruction, Z-stacks were collected under an Olympus confocal microscope and processed using ImageJ software (see Text S1 for details).

### Western Blotting and Zymography

Standard Western blot procedures were employed, using Bio-Rad eletrophoresis and transfer apparatus. Gelatin zymography was performed using purified human pro-MMP-2 (Calbiochem) as well as neonatal lung lysates as standards.

### Cortical Cell Culture

Dissociated cortical cells from BAT-lacZ mice were cultured overnight. ECs and radial glia were identified by immunostaining, while lacZ expression was analyzed by X-gal reaction (see Text S1 for details).

### Quantitative Analysis

Vessel length and branching frequency from every fourth of 50 µm coronal sections of each brain were manually quantified using Nikon NIS-Elements BR 3.0 software. For BAT-lacZ expression and pericyte density analysis, total numbers of lacZ^+^ ECs or PDGFRβ^+^ pericytes were counted and normalized against total vessel length. At least three brains were used for each genotype under each condition, and matching sections were used for controls and mutants. Statistics was performed using Student's *t* test when involving two conditions, and ANOVA followed by Tukey's post hoc test when involving more conditions. All data are represented as mean ± SEM.

## Supporting Information

Figure S1 Generation of *orc3* conditional knockout allele. (A) Schematic diagram of generation of the *orc3* conditional allele. To generate the *orc3* conditional allele, a loxP site is inserted in the intron between exons 4 and 5 and between exons 7 and 8. (B) Southern blot analysis of BAC clones after recombination in bacteria. (C) PCR genotyping of the *orc3* conditional allele. (D) Western analysis of Orc3 expression. E13.5 *orc3/emx1-cre* mutant brains (mt) showed severely reduced Orc3 expression in comparison to controls (ctrl, *orc3* homozygotes). Tubulin was used as loading control. (E) Quantification of Orc3 Western results. Averaging of three independent sets of results showed an 84% loss of Orc3 protein in mutant brains.
(TIF)

Figure S2 Specific targeting of neural cell types by *nestin-cre*. An mTomato/mEGFP double fluorescent *cre* reporter was introduced into the *nestin-cre* background. At E17.5, extensive recombination and mEGFP (in green) expression was observed in cortical radial glia, neurons, and thalamo-cortical axons (A). By contrast, the entire blood vessel network remains untargeted and continues to express mTomato (in red) (B). Cell nuclei were counterstained using DAPI (C). Scale bar (in D): 500 µm for all panels.
(TIF)

Figure S3 Analysis of Pax6 protein expression in E16.5 cortices by Western blotting. (A) Western blot analysis of Pax6 protein levels in *orc3/nestin-cre* control and mutant cortices at E16.5. The overall level of Pax6 protein appears substantially reduced in mutants, in contrast to that of Tubulin (Tub). (B) Quantitative analysis of Pax6 protein expression. The level of Pax6 protein in the whole cortex is reduced by ∼41% in mutants. However, after normalizing against radial glial cell numbers, analysis showed that the expression level of Pax6 in individual cells is not significantly changed in mutants.
(TIF)

Figure S4 Defective cortical neuron migration but normal neuronal fate specification and progenitor marker expression in *orc3/nestin-cre* mutants. (A–B) Nissl staining of P12 control and mutant brains. Severe reduction in cortical thickness was observed in mutants (B), as compared to controls (A). (C–F) Analysis of cortical neuron migration by BrdU birthdating. Early born neurons were labeled by BrdU injection at E12.5 and analyzed at P0 (C–D). Relatively normal localization to deep layers was observed in mutants. Late born neurons were labeled by BrdU injection at E15.5 and analyzed at P0 (E–F). Delays in migration were observed for large numbers of BrdU positive cells in the mutant cortex, where they appeared concentrated at the bottom of the cortical wall (arrowhead in F). (G–H) Quantification of neuronal migration. Neonatal cortices were divided into 10 horizontal subdivisions and the fractions of BrdU positive cells in each subdivision were determined and compared between control and mutant samples (error bars are standard deviation; * *p*<0.05; ** *p*<0.01; *** *p*<0.005; *n* = 3 for all; Student's *t* test). (I–N) Normal cell fate specification of cortical neurons. Upper layer neurons were stained for Cux1 (I–J) and deep layer neurons were stained for Ctip2 (K–L) at P0. Despite defective radial migration, mutant neurons are properly specified and express normal levels of Cux1 and Ctip2. Normal Tbr1 expression was also observed in mutants as compared to controls at E16.5 (M–N). (O–P) Normal cell fate specification of GABAergic interneurons in the ventral forebrain. Interneurons were stained for Dlx1 expression at E15.5. Normal expression was observed in the developing striatum as well as along the two main migratory streams in the cortex. (Q–R) Normal cell fate specification of intermediate progenitors. No significant differences were observed between controls and mutants for the intermediate progenitor marker Tbr2 at E15.5. See quantification in (W). (S–V) Normal expression of layer-specific neuronal markers Cux1 (S and T) and Ctip2 (U and V) at E16.5. No obvious differences were observed between controls and mutants. See quantification in (X). (W) Quantification of Tbr2 positive cell numbers in the ventricular/subventricular zone at E15.5. No significant differences were observed between controls and mutants at E15.5 (*p* = 0.44, *n* = 7), although a significant reduction was observed at E16.5 (see Figure 2). (X) Quantification of Cux1 and Ctip2 positive cell numbers in the cortex at E16.5. No significant differences were observed between controls and mutants in the numbers of either Cux1 or Ctip2 positive cells (Cux1: *p* = 0.7, *n* = 6; Ctip2: *p* = 0.42, *n* = 6). Scale bar in (V): 400 µm for (C–F), 200 µm for (I–N), 500 µm for (O–P), and 100 µm for (Q–V).
(TIF)

Figure S5 Normal vessel development in the hindbrain and the spinal cord of *orc3/nestin-cre* mutants. (A–B) IB4 staining (in green) of coronal sections of control (A) and mutant (B) hindbrains at P0. (C) Quantification of vessel density in the hindbrain at P0. No significant differences were observed between controls and mutants (*p* = 0.37, *n* = 10). (D) Quantification of vessel density in the spinal cord at P0. No significant differences were observed between controls and mutants (*p* = 0.98, *n* = 4). Scale bar in (A): 500 µm for (A–B).
(TIF)

Figure S6 Effects of *hGFAP-cre*–mediated radial glial ablation and effects of defective neuronal migration and production on cortical vessel development. (A–B) Effects of *hGFAP-cre*–mediated *orc3* deletion on cortical angiogenesis. Ablation of radial glia by *hGFAP-cre* results in defective cortical vessel development near the midline (arrows). The lesser phenotypic severity is likely due to the later onset of *hGFAP-cre* than *nestin-cre* expression. (C–D) Quantification of vessel density and branching frequency in the medial cortex of *orc3/hGFAP-cre* control and mutant neonates. Significant decreases were observed in both vessel density (*p* = 5.25×10^−8^; *n* = 6) and branching frequency (*p* = 8.48×10^−6^; *n* = 6) in mutant cortices. (E–H) Effects of *β1 integrin/emx1-cre* and *brca1/emx1-cre* single and *β1 integrin*/*brca1/emx1-cre* double (dko) mutation on cortical angiogenesis. Although vessel growth pattern is altered in *β1 integrin/emx1-cre* single and *β1 integrin*/*brca1/emx1-cre* double mutants, vessel density does not appear consistently altered in mutant neonates. This suggests that defective cortical neuron migration and production is unlikely a major factor in vessel regression following neural progenitor ablation. (I–J) Quantification of neonatal brain vessel density and branching frequency. No significant differences were observed between control and *β1 integrin/emx1-cre* and *brca1/emx1-cre* single or double mutants (*** *p*<0.001; *ns*, *p*>0.05 for all; *n* = 3 for each genotype). Scale bar in (A): 500 µm for (A–B) and (E–H).
(TIF)

Figure S7 Expression levels of VEGF A isoform and angiopoietin-1 mRNA are not affected in *orc3/nestin-cre* mutant cortices at E16.5. qRT-PCR analysis of three VEGF A isoform and angiopoietin-1 mRNA expression in control and mutant cortices at E16.5 showed no significant differences for any of these mRNAs (*p*>0.4 and *n* = 4 for all).
(TIF)

Figure S8 BAT-lacZ expression along cortical vessels during normal development and comparison of cortical Wnt ligand and inhibitor expression in *orc3/nestin-cre* controls and mutants. (A–D) X-gal and IB4 double staining of E15.5 (A), E16.5 (B), E17.5 (C), and P0 (D) cortical plate vessels in wild-type animals. lacZ positive ECs are highlighted by arrowheads. Note strong expression in some upper layer neurons at P0 (D). (E) Quantification of lacZ positive EC density in E14.5-P0 cortical plates. Significant differences are observed between all consecutive stages (*p*<0.003), except between E15.5 and E16.5 (*p* = 0.53). (F) qRT-PCR analysis of Wnt ligand and extracellular inhibitor expression in control and mutant cortices at E16.5. No significant differences were observed for either the Wnt ligands wnt7a and wnt7b or the inhibitors sfrp1 sfrp2, wif1, and dkk1 (*p*>0.4 and *n* = 4). Scale bar in (A): 600 µm for (A–D).
(TIF)

Figure S9 No significant numbers of microglia appear in the mutant cortical plate until birth. (A–B) Staining against Cd11b (in red) at E16.5 showed no significant accumulation of microglia in either the control (A) or the mutant (B) cortical plate. Nuclei were counterstained with DAPI (in blue). (C–D) Staining against Cd11b (in red) at P0 showed a significant number of microglia in the cortical plate of mutants (D), in contrast to that of controls (C). Scale bar in (A): 100 µm for (A–D).
(TIF)

Figure S10 LiCl treatment activates Wnt signaling, Glut-1 expression, and EC proliferation in cortical vessels but has no effects on noncortical vessel development. (A–C) Up-regulation of BAT-lacZ reporter in cortical ECs following LiCl treatment. The density of BAT-lacZ positive ECs was substantially increased (control, 3.1±0.2/mm; LiCl treated, 4.8±0.3/mm; *p* = 0.007, *n* = 3). (D–F) Up-regulation of Glut-1 expression in cortical ECs following LiCl treatment. Cortical plate is indicated by pairs of white bars in (D and E). The intensity of Glut-1 staining was significantly increased after treatment (*p* = 2.5×10^−6^, *n* = 10). (G–I) Increased proliferation of cortical ECs following LiCl treatment. The density of BrdU positive ECs was significantly increased (*p* = 0.03, *n* = 11). (J–L) Vessel density is not significantly changed in E18.5 striatum, following LiCl treatment at E16.5 and E17.5 (NaCl treated, 18,319±1,146 µm/mm^2^; LiCl treated, 18,553±882 µm/mm^2^; *p* = 0.88, *n* = 4). (M–O) Vessel density is not significantly changed in E18.5 heart, following LiCl treatment at E16.5 and E17.5 (NaCl treated, 27,409±1,379 µm/mm^2^; LiCl treated, 25,943±1,071 µm/mm^2^; *p* = 0.45, *n* = 3). Scale bar in (N): 150 µm for (A–B), 200 µm for (D–E), 100 µm for (G–H), and 200 µm for (J–N).
(TIF)

Figure S11 Normal expression of Wnt ligands and inhibitors as well as layer-specific neuronal markers after LiCl treatment. (A) qRT-PCR analysis of Wnt ligand and extracellular inhibitor expression in control and LiCl-treated cortices at E16.5. No significant differences were observed for either the Wnt ligands wnt7a and wnt7b or the inhibitors sfrp1 sfrp2, wif1, and dkk1 (*p*>0.58 and *n* = 4 for all). (B–G) Expression of layer-specific neuronal markers Cux1 (B, D, F) and Ctip2 (C, E, G) in the cortex of control (B–C), LiCl (D–E), or SB216763-treated (F–G) embryos. No obvious differences were observed between control and treated brains for either marker. (H–I) Quantification of Cux1 and Ctip2 positive neuron density in control, LiCl, and SB216763 (SB) treated brains at E17.5. ANOVA showed no significant differences in either Cux1 or Ctip2 positive neuronal density between any of the groups (*p*>0.4, *n* = 3 for each). Scale bar in (C): 100 µm for (B–G).
(TIF)

Figure S12 Analysis of metalloproteinase expression in *orc3/nestin-cre* mutant cortex and confirmation of Timp2 requirement in pro-MMP2 activation in vivo. (A) RT-PCR analysis of MT1-MMP and MMP-2 expression in the control and mutant cortex at E16.5. No significant differences were observed for MT1-MMP, but MMP-2 mRNA level is significantly increased in mutants. (B) Confirmation of Timp2 requirement in pro-MMP2 activation in vivo. Neonatal lungs were used because of normally high levels of pro-MMP-2 activation (cleaved MMP-2) at this stage. In *Timp2* homozygous mutants, cleaved MMP-2 is completely absent (asterisk).
(TIF)

Movie S1 Three-dimensional reconstruction by confocal microscopy of interactions between ECs, pericytes, and radial glial fibers along one segment of blood vessels in the E16.5 mouse cortex. ECs, pericytes, and radial glia were labeled with IB4 (in green) and NG2 (in blue) and GLAST (in red) antibodies, respectively.
(MP4)

Movie S2 Three-dimensional reconstruction of interactions between ECs, pericytes, and radial glial fibers along another segment of blood vessels in the E16.5 mouse cortex. ECs, pericytes, and radial glia were labeled as in Movie S1.
(MP4)

Table S1 Summary of effects of *orc3/nestin-cre* mutation on cortical neurogenesis.
(DOC)

Text S1 Supplemental experimental procedures.
(DOC)

## Abbreviations

CNS: central nervous system
EC: endothelial cell
GSK-3β: glycogen synthase kinase 3β
IB4: isolectin B4
MMP: matrix metalloproteinase
ORC: origin recognition complex
PH3: phospho-histone 3
VEGF: vascular endothelial growth factor
