# Supplementary figures and images for: Radial Glial Neural Progenitors Regulate Nascent Brain Vascular Network Stabilization Via Inhibition of Wnt Signaling
Source: PLoS Biol. 2013 Jan 22;11(1):e1001469. doi: 10.1371/journal.pbio.1001469 (PMC3551952; doi:10.1371/journal.pbio.1001469)

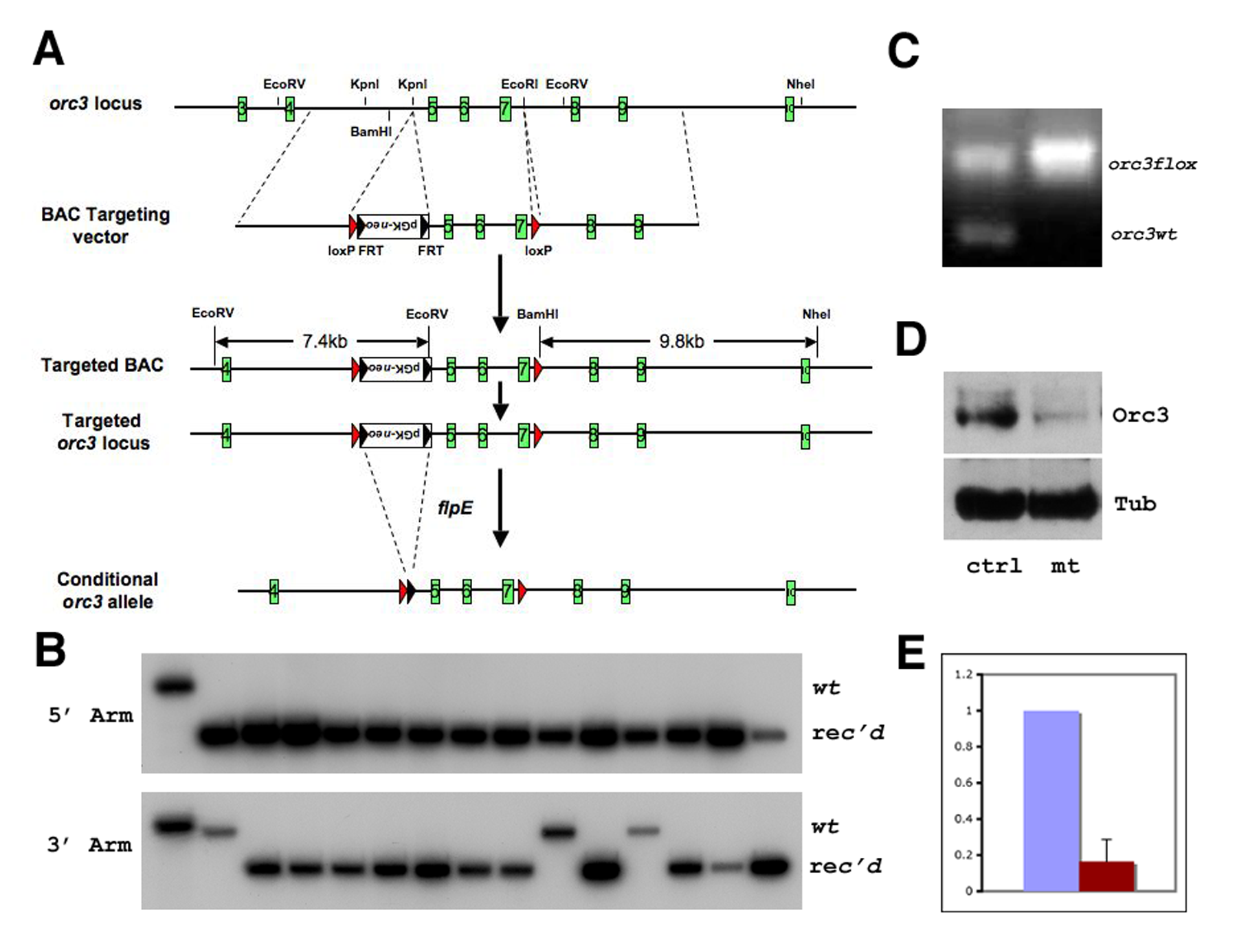

Supplement: Figure S1 — Generation of orc3 conditional knockout allele. (A) Schematic diagram of generation of the orc3 conditional allele. To generate the orc3 conditional allele, a loxP site is inserted in the intron between exons 4 and 5 and between exons 7 and 8. (B) Southern blot analysis of BAC clones after recombination in bacteria. (C) PCR genotyping of the orc3 conditional allele. (D) Western analysis of Orc3 expression. E13.5 orc3/emx1-cre mutant brains (mt) showed severely reduced Orc3 expression in comparison to controls (ctrl, orc3 homozygotes). Tubulin was used as loading control. (E) Quantification of Orc3 Western results. Averaging of three independent sets of results showed an 84% loss of Orc3 protein in mutant brains. (TIF) [file pbio.1001469.s001.tif]

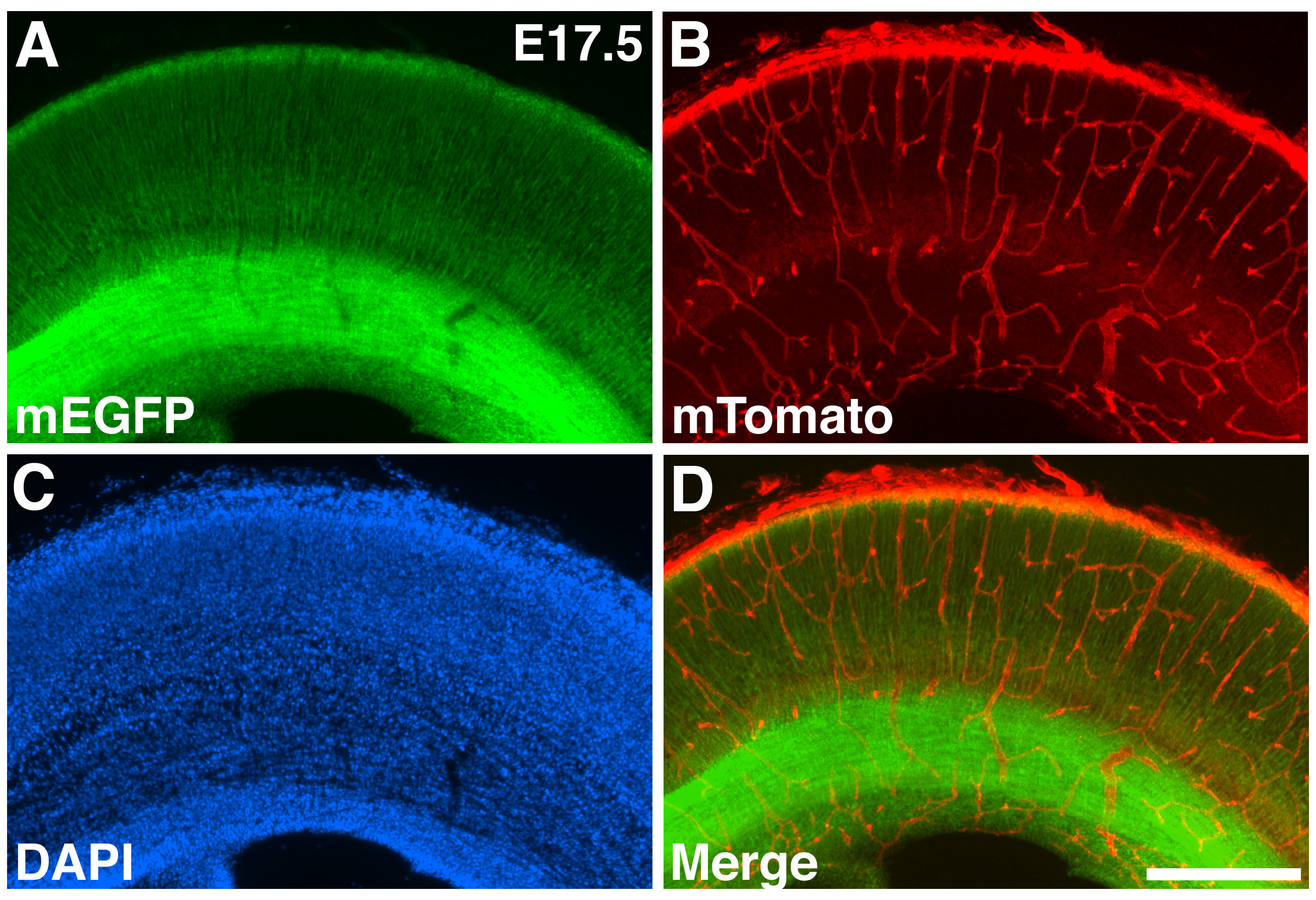

Supplement: Figure S2 — Specific targeting of neural cell types by nestin-cre. An mTomato/mEGFP double fluorescent cre reporter was introduced into the nestin-cre background. At E17.5, extensive recombination and mEGFP (in green) expression was observed in cortical radial glia, neurons, and thalamo-cortical axons (A). By contrast, the entire blood vessel network remains untargeted and continues to express mTomato (in red) (B). Cell nuclei were counterstained using DAPI (C). Scale bar (in D): 500 µm for all panels. (TIF) [file pbio.1001469.s002.tif]

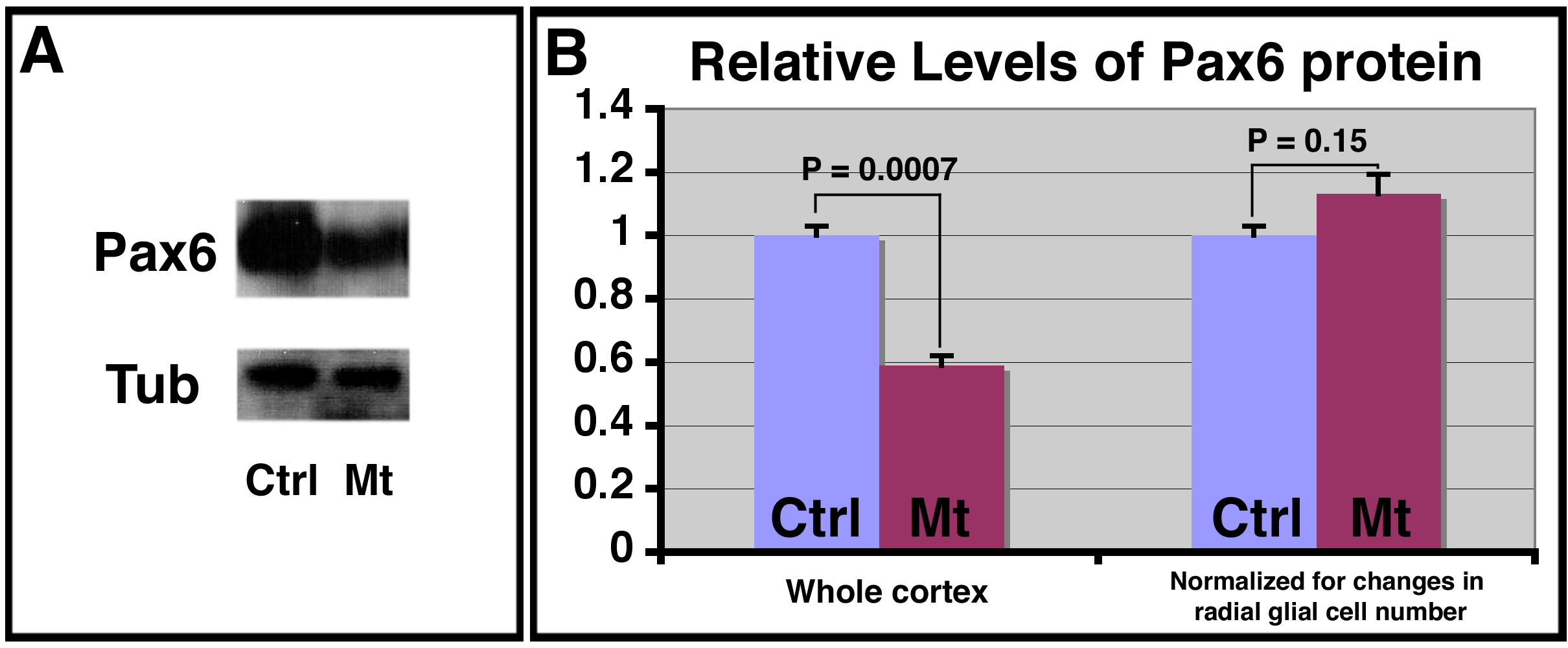

Supplement: Figure S3 — Analysis of Pax6 protein expression in E16.5 cortices by Western blotting. (A) Western blot analysis of Pax6 protein levels in orc3/nestin-cre control and mutant cortices at E16.5. The overall level of Pax6 protein appears substantially reduced in mutants, in contrast to that of Tubulin (Tub). (B) Quantitative analysis of Pax6 protein expression. The level of Pax6 protein in the whole cortex is reduced by ∼41% in mutants. However, after normalizing against radial glial cell numbers, analysis showed that the expression level of Pax6 in individual cells is not significantly changed in mutants. (TIF) [file pbio.1001469.s003.tif]

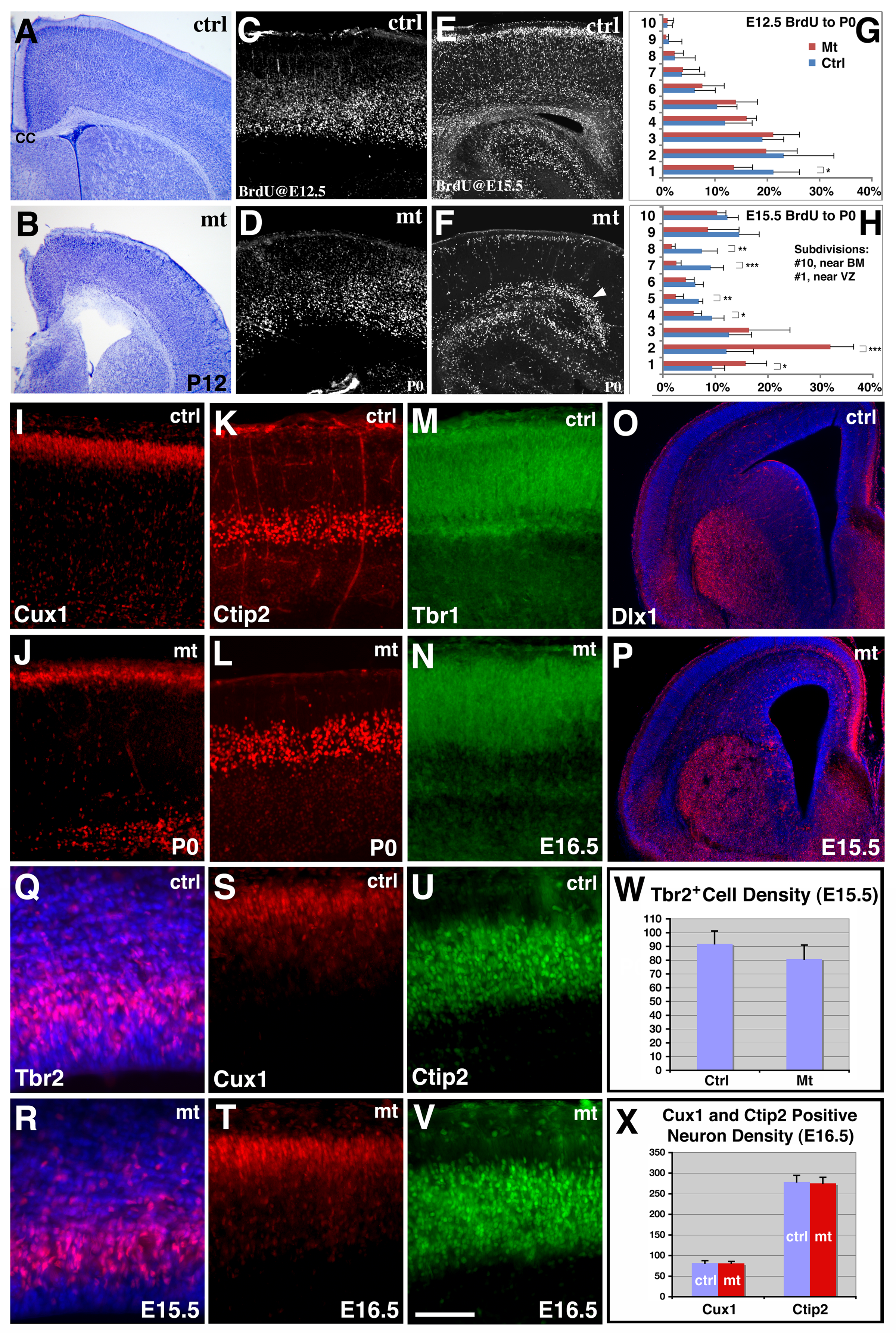

Supplement: Figure S4 — Defective cortical neuron migration but normal neuronal fate specification and progenitor marker expression in orc3/nestin-cre mutants. (A–B) Nissl staining of P12 control and mutant brains. Severe reduction in cortical thickness was observed in mutants (B), as compared to controls (A). (C–F) Analysis of cortical neuron migration by BrdU birthdating. Early born neurons were labeled by BrdU injection at E12.5 and analyzed at P0 (C–D). Relatively normal localization to deep layers was observed in mutants. Late born neurons were labeled by BrdU injection at E15.5 and analyzed at P0 (E–F). Delays in migration were observed for large numbers of BrdU positive cells in the mutant cortex, where they appeared concentrated at the bottom of the cortical wall (arrowhead in F). (G–H) Quantification of neuronal migration. Neonatal cortices were divided into 10 horizontal subdivisions and the fractions of BrdU positive cells in each subdivision were determined and compared between control and mutant samples (error bars are standard deviation; * p<0.05; ** p<0.01; *** p<0.005; n = 3 for all; Student's t test). (I–N) Normal cell fate specification of cortical neurons. Upper layer neurons were stained for Cux1 (I–J) and deep layer neurons were stained for Ctip2 (K–L) at P0. Despite defective radial migration, mutant neurons are properly specified and express normal levels of Cux1 and Ctip2. Normal Tbr1 expression was also observed in mutants as compared to controls at E16.5 (M–N). (O–P) Normal cell fate specification of GABAergic interneurons in the ventral forebrain. Interneurons were stained for Dlx1 expression at E15.5. Normal expression was observed in the developing striatum as well as along the two main migratory streams in the cortex. (Q–R) Normal cell fate specification of intermediate progenitors. No significant differences were observed between controls and mutants for the intermediate progenitor marker Tbr2 at E15.5. See quantification in (W). (S–V) Normal expression of la [file pbio.1001469.s004.tif]

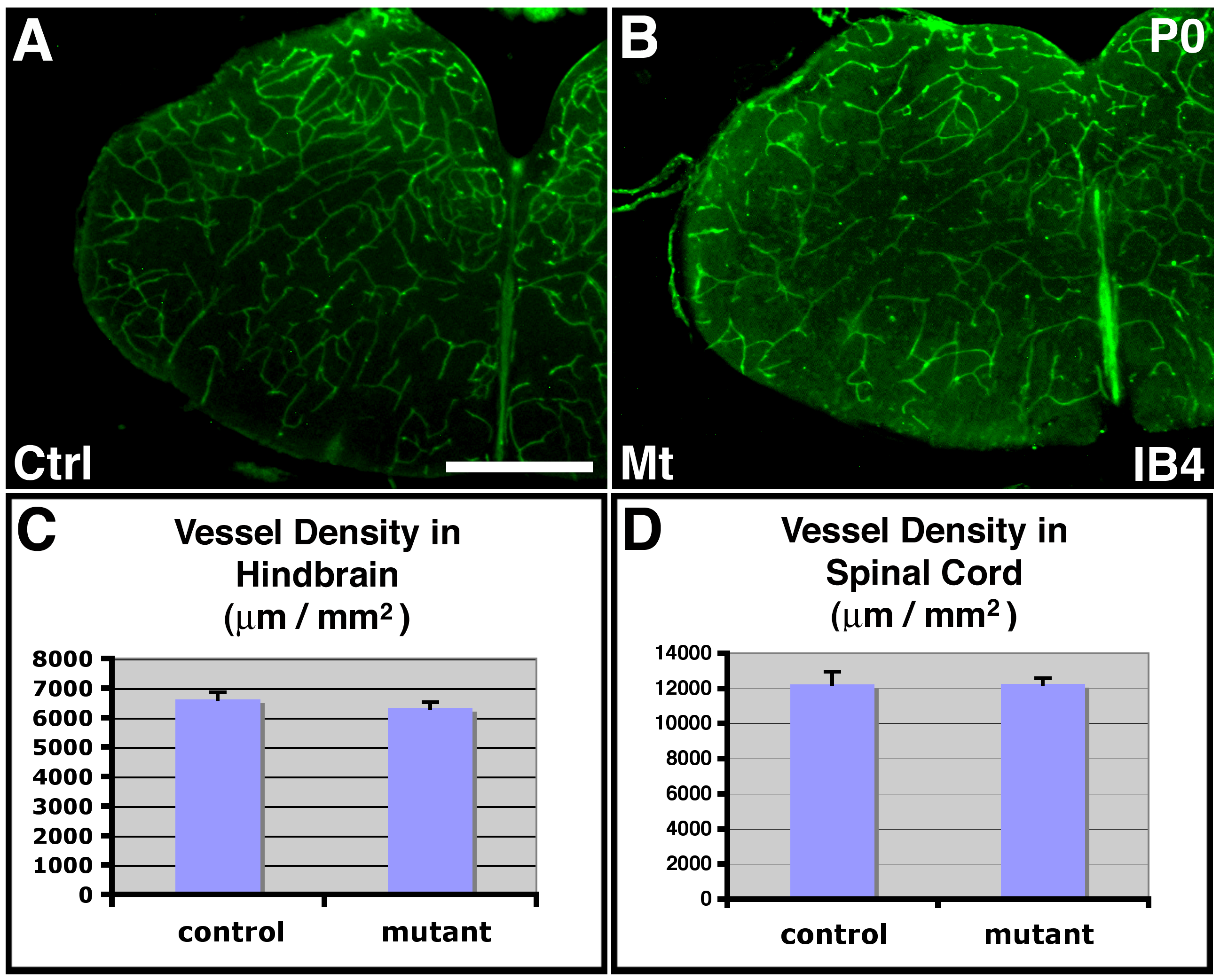

Supplement: Figure S5 — Normal vessel development in the hindbrain and the spinal cord of orc3/nestin-cre mutants. (A–B) IB4 staining (in green) of coronal sections of control (A) and mutant (B) hindbrains at P0. (C) Quantification of vessel density in the hindbrain at P0. No significant differences were observed between controls and mutants (p = 0.37, n = 10). (D) Quantification of vessel density in the spinal cord at P0. No significant differences were observed between controls and mutants (p = 0.98, n = 4). Scale bar in (A): 500 µm for (A–B). (TIF) [file pbio.1001469.s005.tif]

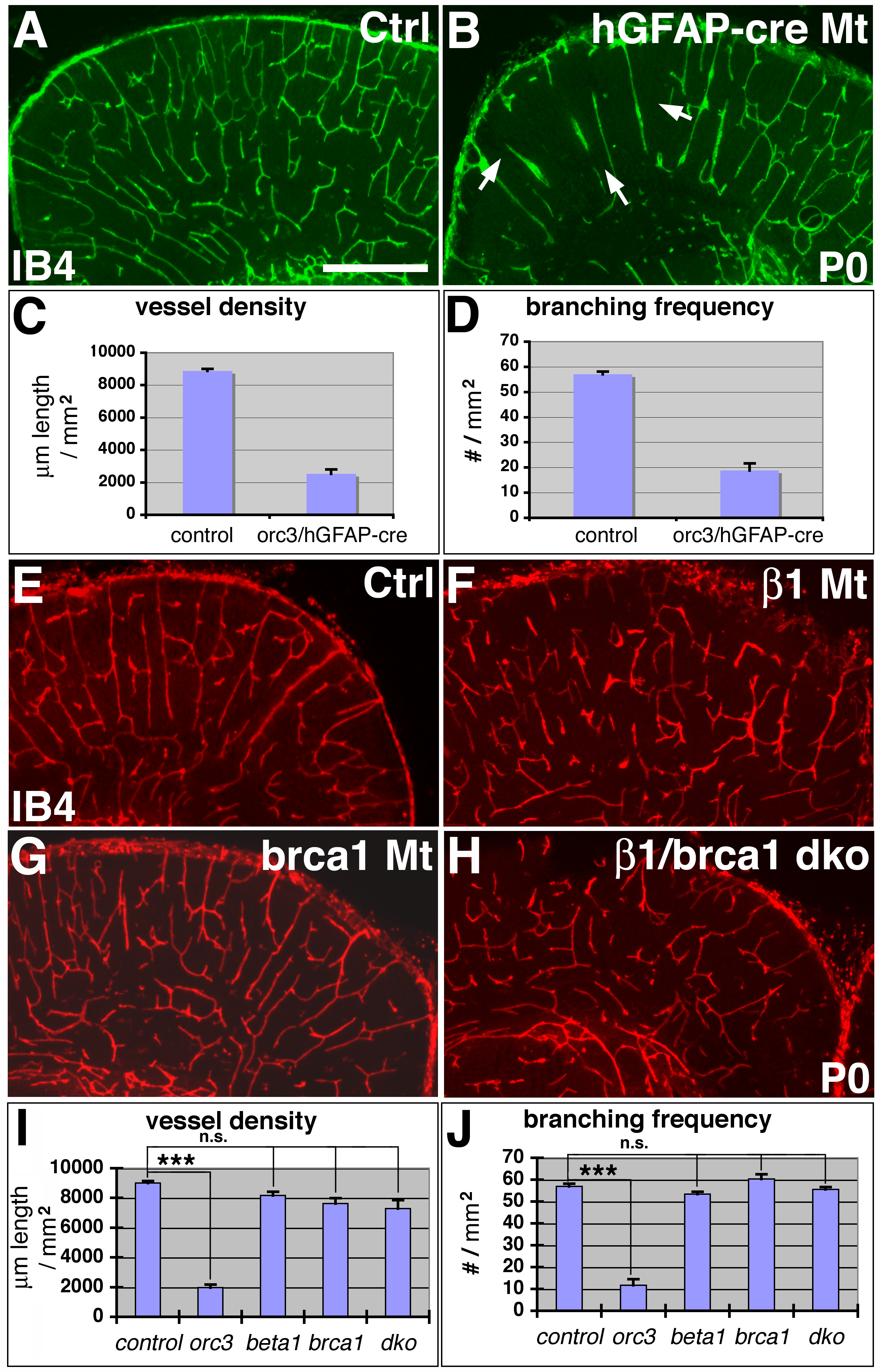

Supplement: Figure S6 — Effects of hGFAP-cre–mediated radial glial ablation and effects of defective neuronal migration and production on cortical vessel development. (A–B) Effects of hGFAP-cre–mediated orc3 deletion on cortical angiogenesis. Ablation of radial glia by hGFAP-cre results in defective cortical vessel development near the midline (arrows). The lesser phenotypic severity is likely due to the later onset of hGFAP-cre than nestin-cre expression. (C–D) Quantification of vessel density and branching frequency in the medial cortex of orc3/hGFAP-cre control and mutant neonates. Significant decreases were observed in both vessel density (p = 5.25×10^−8^; n = 6) and branching frequency (p = 8.48×10^−6^; n = 6) in mutant cortices. (E–H) Effects of β1 integrin/emx1-cre and brca1/emx1-cre single and β1 integrin/brca1/emx1-cre double (dko) mutation on cortical angiogenesis. Although vessel growth pattern is altered in β1 integrin/emx1-cre single and β1 integrin/brca1/emx1-cre double mutants, vessel density does not appear consistently altered in mutant neonates. This suggests that defective cortical neuron migration and production is unlikely a major factor in vessel regression following neural progenitor ablation. (I–J) Quantification of neonatal brain vessel density and branching frequency. No significant differences were observed between control and β1 integrin/emx1-cre and brca1/emx1-cre single or double mutants (*** p<0.001; ns, p>0.05 for all; n = 3 for each genotype). Scale bar in (A): 500 µm for (A–B) and (E–H). (TIF) [file pbio.1001469.s006.tif]

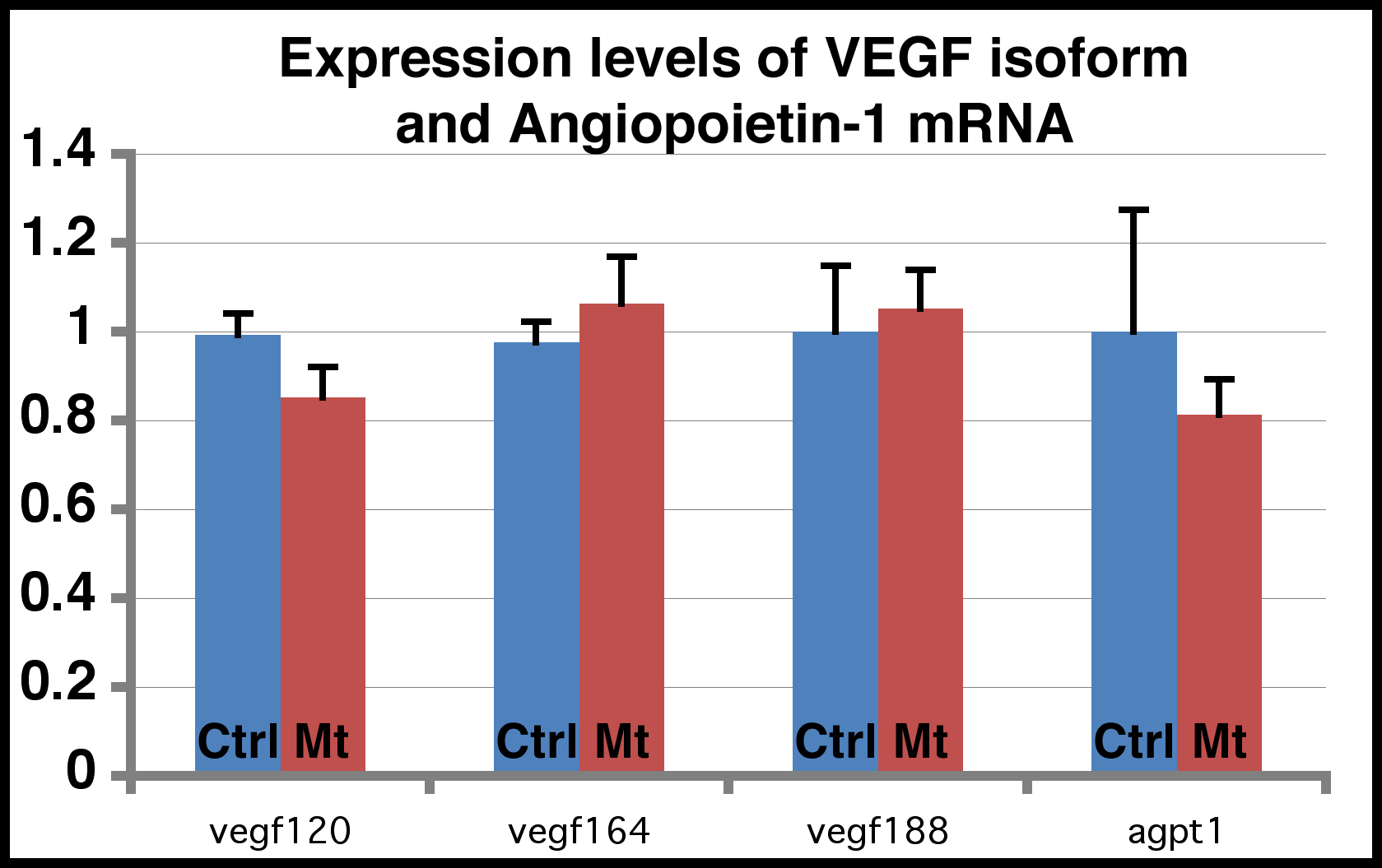

Supplement: Figure S7 — Expression levels of VEGF A isoform and angiopoietin-1 mRNA are not affected in orc3/nestin-cre mutant cortices at E16.5. qRT-PCR analysis of three VEGF A isoform and angiopoietin-1 mRNA expression in control and mutant cortices at E16.5 showed no significant differences for any of these mRNAs (p>0.4 and n = 4 for all). (TIF) [file pbio.1001469.s007.tif]

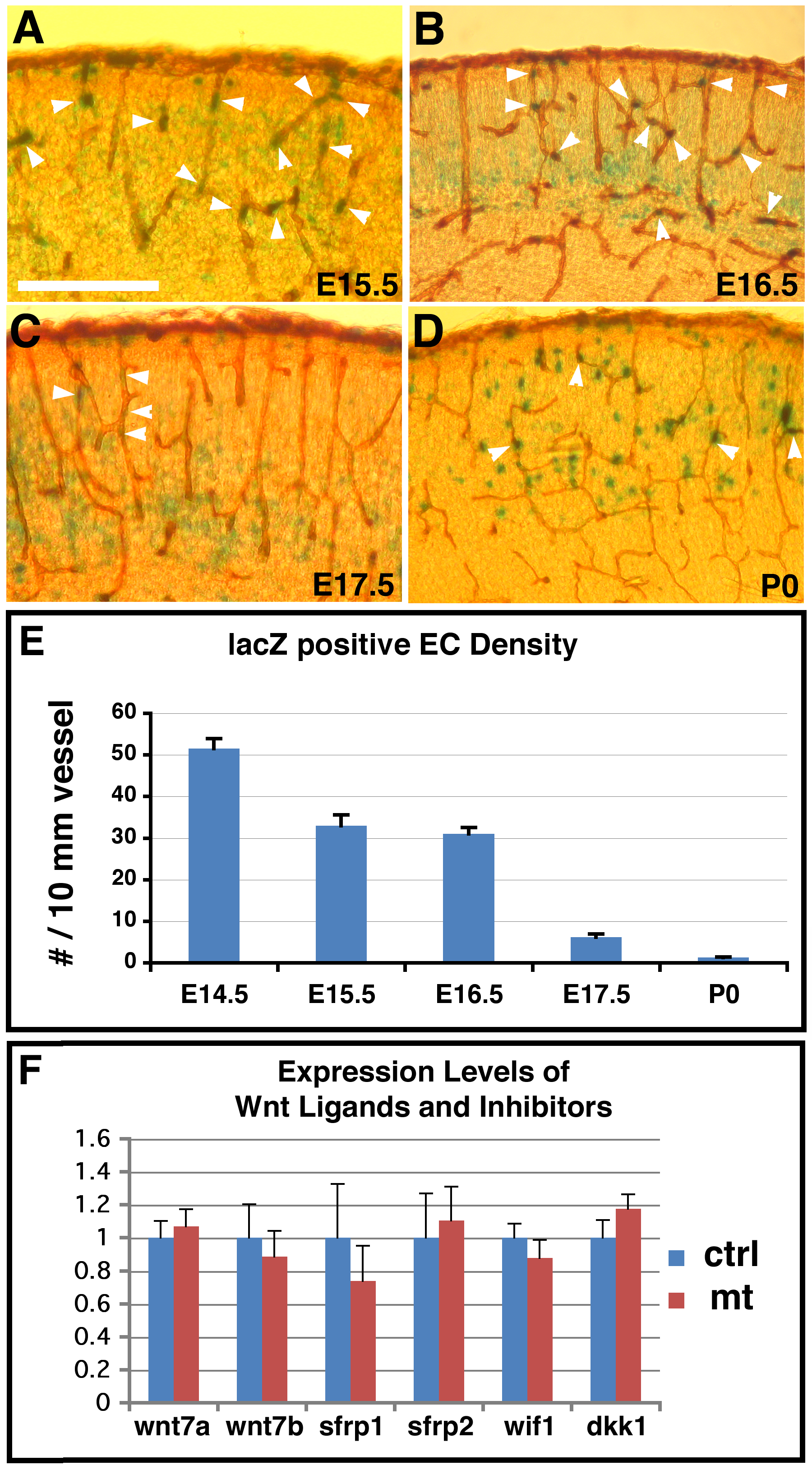

Supplement: Figure S8 — BAT-lacZ expression along cortical vessels during normal development and comparison of cortical Wnt ligand and inhibitor expression in orc3/nestin-cre controls and mutants. (A–D) X-gal and IB4 double staining of E15.5 (A), E16.5 (B), E17.5 (C), and P0 (D) cortical plate vessels in wild-type animals. lacZ positive ECs are highlighted by arrowheads. Note strong expression in some upper layer neurons at P0 (D). (E) Quantification of lacZ positive EC density in E14.5-P0 cortical plates. Significant differences are observed between all consecutive stages (p<0.003), except between E15.5 and E16.5 (p = 0.53). (F) qRT-PCR analysis of Wnt ligand and extracellular inhibitor expression in control and mutant cortices at E16.5. No significant differences were observed for either the Wnt ligands wnt7a and wnt7b or the inhibitors sfrp1 sfrp2, wif1, and dkk1 (p>0.4 and n = 4). Scale bar in (A): 600 µm for (A–D). (TIF) [file pbio.1001469.s008.tif]

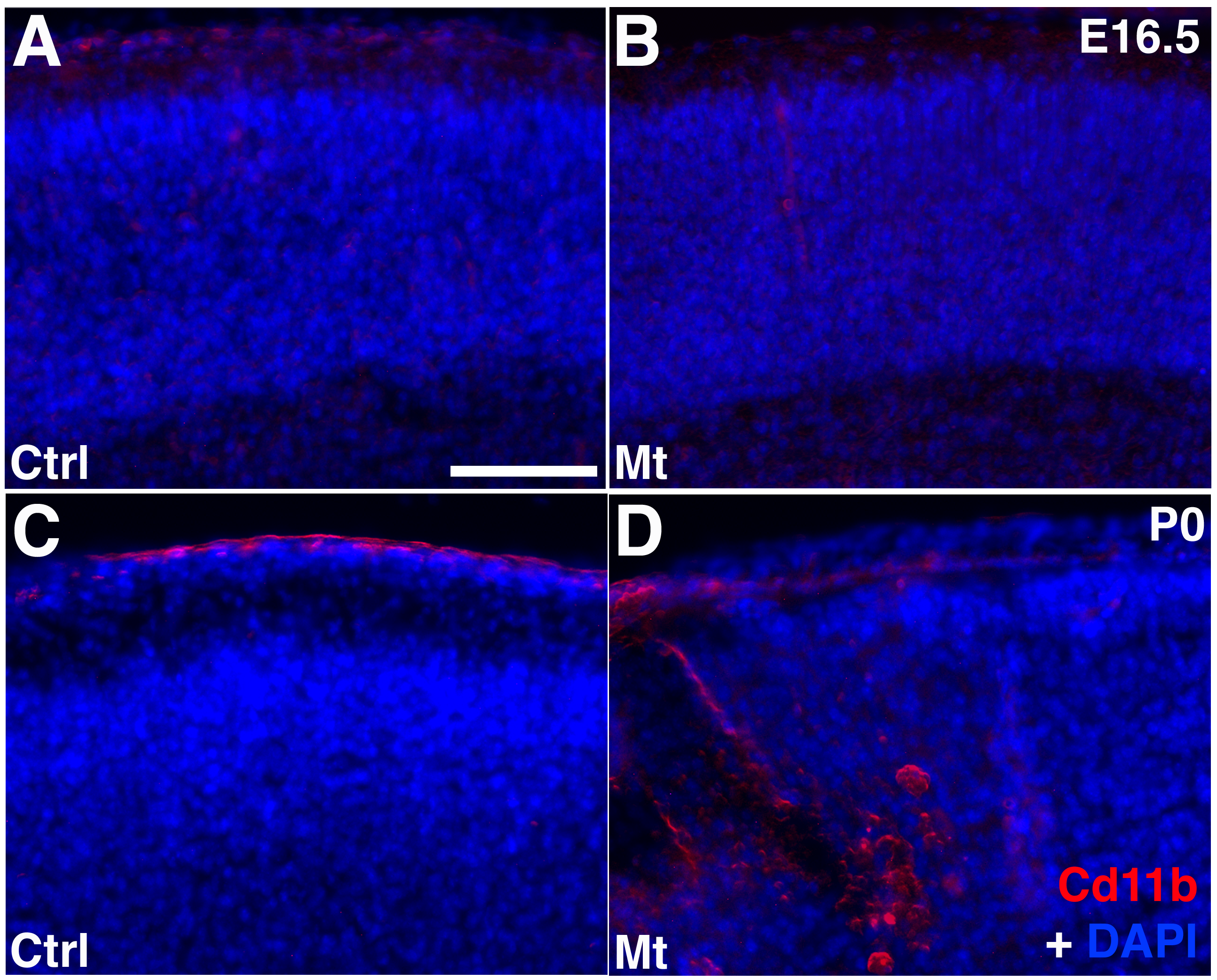

Supplement: Figure S9 — No significant numbers of microglia appear in the mutant cortical plate until birth. (A–B) Staining against Cd11b (in red) at E16.5 showed no significant accumulation of microglia in either the control (A) or the mutant (B) cortical plate. Nuclei were counterstained with DAPI (in blue). (C–D) Staining against Cd11b (in red) at P0 showed a significant number of microglia in the cortical plate of mutants (D), in contrast to that of controls (C). Scale bar in (A): 100 µm for (A–D). (TIF) [file pbio.1001469.s009.tif]

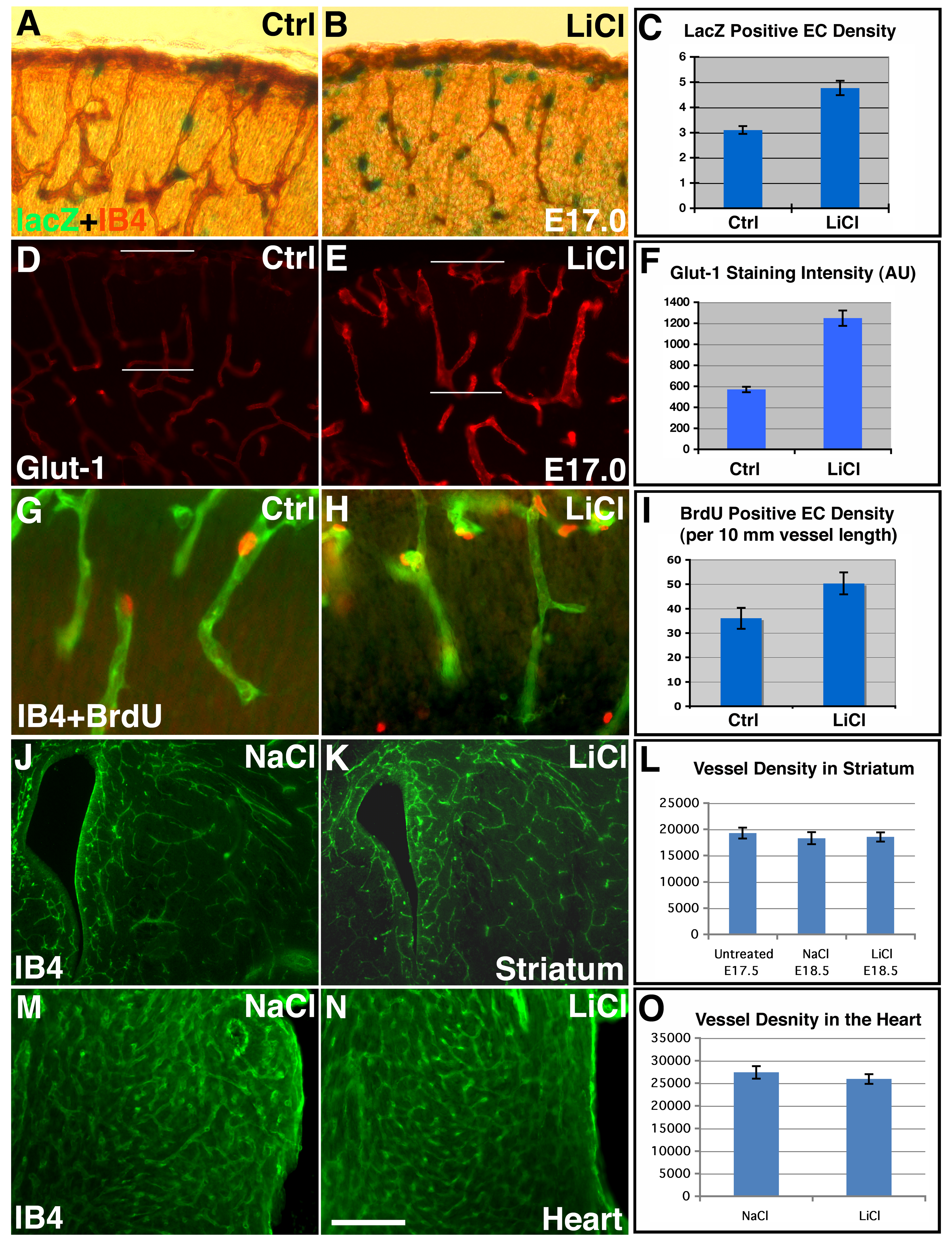

Supplement: Figure S10 — LiCl treatment activates Wnt signaling, Glut-1 expression, and EC proliferation in cortical vessels but has no effects on noncortical vessel development. (A–C) Up-regulation of BAT-lacZ reporter in cortical ECs following LiCl treatment. The density of BAT-lacZ positive ECs was substantially increased (control, 3.1±0.2/mm; LiCl treated, 4.8±0.3/mm; p = 0.007, n = 3). (D–F) Up-regulation of Glut-1 expression in cortical ECs following LiCl treatment. Cortical plate is indicated by pairs of white bars in (D and E). The intensity of Glut-1 staining was significantly increased after treatment (p = 2.5×10^−6^, n = 10). (G–I) Increased proliferation of cortical ECs following LiCl treatment. The density of BrdU positive ECs was significantly increased (p = 0.03, n = 11). (J–L) Vessel density is not significantly changed in E18.5 striatum, following LiCl treatment at E16.5 and E17.5 (NaCl treated, 18,319±1,146 µm/mm2; LiCl treated, 18,553±882 µm/mm2; p = 0.88, n = 4). (M–O) Vessel density is not significantly changed in E18.5 heart, following LiCl treatment at E16.5 and E17.5 (NaCl treated, 27,409±1,379 µm/mm2; LiCl treated, 25,943±1,071 µm/mm2; p = 0.45, n = 3). Scale bar in (N): 150 µm for (A–B), 200 µm for (D–E), 100 µm for (G–H), and 200 µm for (J–N). (TIF) [file pbio.1001469.s010.tif]

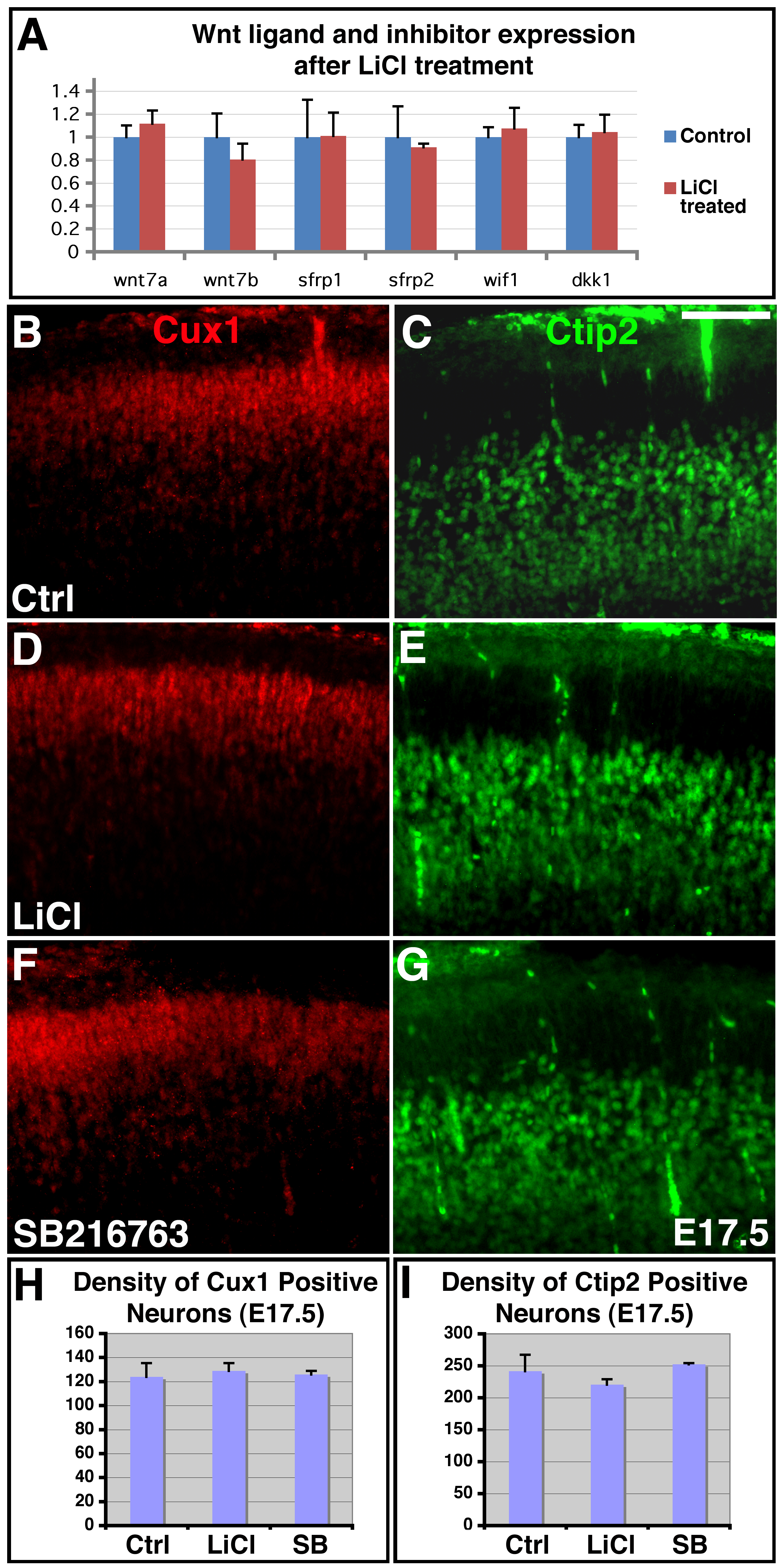

Supplement: Figure S11 — Normal expression of Wnt ligands and inhibitors as well as layer-specific neuronal markers after LiCl treatment. (A) qRT-PCR analysis of Wnt ligand and extracellular inhibitor expression in control and LiCl-treated cortices at E16.5. No significant differences were observed for either the Wnt ligands wnt7a and wnt7b or the inhibitors sfrp1 sfrp2, wif1, and dkk1 (p>0.58 and n = 4 for all). (B–G) Expression of layer-specific neuronal markers Cux1 (B, D, F) and Ctip2 (C, E, G) in the cortex of control (B–C), LiCl (D–E), or SB216763-treated (F–G) embryos. No obvious differences were observed between control and treated brains for either marker. (H–I) Quantification of Cux1 and Ctip2 positive neuron density in control, LiCl, and SB216763 (SB) treated brains at E17.5. ANOVA showed no significant differences in either Cux1 or Ctip2 positive neuronal density between any of the groups (p>0.4, n = 3 for each). Scale bar in (C): 100 µm for (B–G). (TIF) [file pbio.1001469.s011.tif]

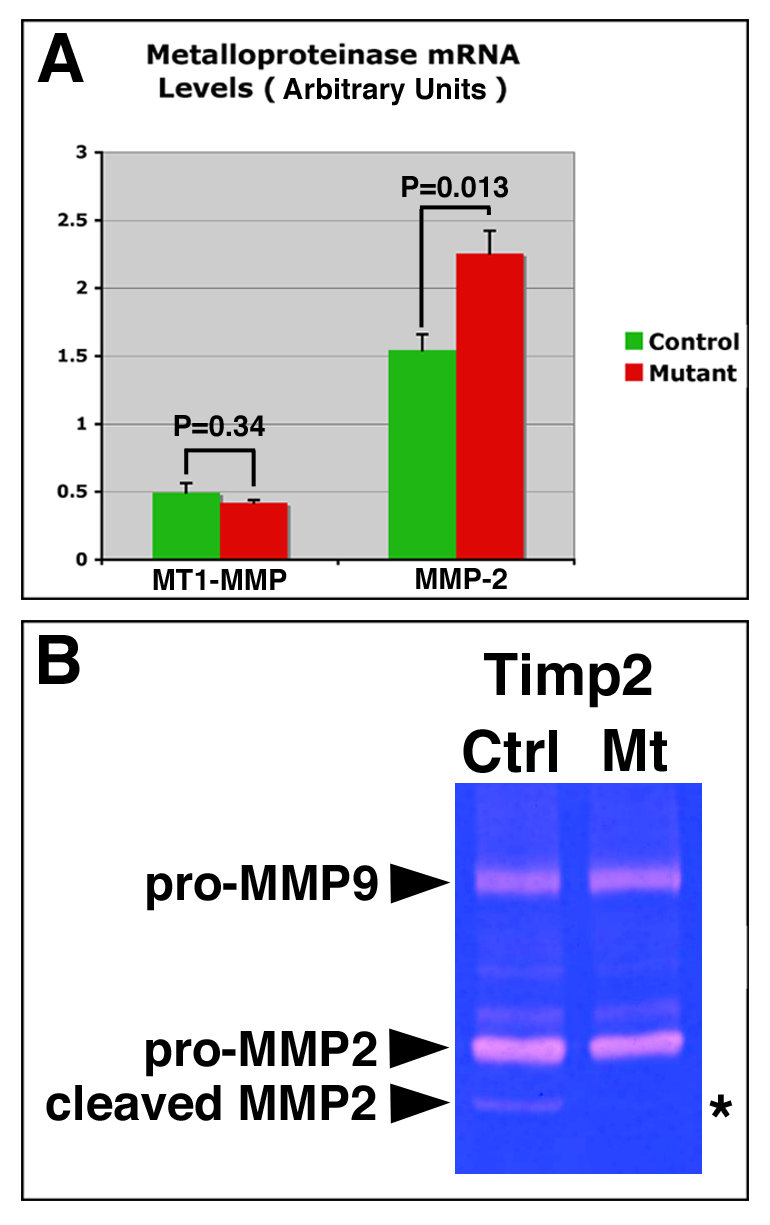

Supplement: Figure S12 — Analysis of metalloproteinase expression in orc3/nestin-cre mutant cortex and confirmation of Timp2 requirement in pro-MMP2 activation in vivo. (A) RT-PCR analysis of MT1-MMP and MMP-2 expression in the control and mutant cortex at E16.5. No significant differences were observed for MT1-MMP, but MMP-2 mRNA level is significantly increased in mutants. (B) Confirmation of Timp2 requirement in pro-MMP2 activation in vivo. Neonatal lungs were used because of normally high levels of pro-MMP-2 activation (cleaved MMP-2) at this stage. In Timp2 homozygous mutants, cleaved MMP-2 is completely absent (asterisk). (TIF) [file pbio.1001469.s012.tif]
