# Supplementary material for: Radial Glial Neural Progenitors Regulate Nascent Brain Vascular Network Stabilization Via Inhibition of Wnt Signaling
Source: PLoS Biol. 2013 Jan 22;11(1):e1001469. doi: 10.1371/journal.pbio.1001469 (PMC3551952; doi:10.1371/journal.pbio.1001469)
Supplement: Table S1 — Summary of effects of orc3/nestin-cre mutation on cortical neurogenesis. (DOC) [file pbio.1001469.s015.doc]

**Table S1. Summary of effects of *orc3/nestin-cre* mutation on cortical neurogenesis**

| Cell type(s) | Marker(s) | Results | Figure panels |
| --- | --- | --- | --- |
| Radial glial progenitors | BrdU | The number of BrdU positive (S-phase) ventricular zone cells is normal at E13.5, but reduced at E15.5 (by ~46%) | Fig. 2A, B (E13.5) Fig. 2C, D (E15.5) |
|  | PH3 | The number of phospho-histone 3 positive (mitotic) radial glial cells is reduced at E16.5 (by ~82%) | Fig. 2I’, J’ |
|  | Ki67 | The number of Ki67 positive cells in the ventricular zone is reduced at E15.5 (by ~24%) | Fig. 2O’, P’ |
|  | RC2 | The density of RC2 positive radial glial fibers is reduced beginning at E15.5 (by ~ 36%), and most severely at P0 | Fig. 2E-F’ (E15.5), Fig. 2G-H’ (P0) |
|  | Pax6 | The number of Pax6 positive radial glial progenitors is reduced at E16.5 (by ~48%) | Fig. 2I, J, K |
| Intermediate progenitors | Tbr2 | The number of Tbr2 positive cells is normal at E15.5, but reduced at E16.5 (by ~78%) | Fig. S3Q, R, W (E15.5), Fig. 2M, N, Q (E16.5) |
| All neural progenitors | Ki67+BrdU | Cell cycle exit by neural progenitors is increased at E15.5 (by ~88%), but unaffected at E16.5 | Fig. 2O-P” (E15.5), R |
| Upper layer neurons | Cux1 | The number of Cux1 positive neurons is normal at E16.5, but reduced at P0 | Fig. S3I, J (P0), Fig. S3S, T, X (E16.5) |
| Deep layer neurons | Ctip2 | The number of Ctip2 positive neurons is normal at both E16.5 and P0 | Fig. S3K, L (P0), Fig. S3U, V, X (E16.5) |
| GABAergic interneurons | Dlx1 | Dlx1 is expressed normally in the ventral forebrain at E15.5 | Fig. S3O, P |
