# Supplementary material for: Radial Glial Neural Progenitors Regulate Nascent Brain Vascular Network Stabilization Via Inhibition of Wnt Signaling
Source: PLoS Biol. 2013 Jan 22;11(1):e1001469. doi: 10.1371/journal.pbio.1001469 (PMC3551952; doi:10.1371/journal.pbio.1001469)
Supplement: Text S1 — Supplemental experimental procedures. (DOC) [file pbio.1001469.s016.doc]

**Supplemental Experimental Procedures**

Molecular biology

BAC recombineering technology was employed for generating the conditional *orc3* allele. Genomic fragments from the *orc3* locus were isolated by BAC insert subcloning followed by colony hybridization and sequencing. Standard molecular biology techniques were employed in vector construction. ES cell clones were screened by Taqman PCR and confirmed by quantitative Southern blotting. Test probes and primers for Taqman PCR are as follows:

Probe 1: 5’CAATACTAAGACCAACTCTTGAGAAATTGGACCTCAA3’;

Primer set 1: 5’AGGACTTTCTAAAAAAGACCCTGGTA3’ and 5’TGTCCTGACTGTGTACAAGTTTTTTAGTAT3’.

Probe 2: 5’AAGTCCTTTTCATGTTATAAGTGCATTCCTTACTTCCTCT3’;

Primer set 2: 5’AAGGGCCTAAACATAGACCTGACA3’ and 5’ATTTCTCAAGAGTTGGTCTTAGTATTGGT3’.

The primer set for genotyping the *orc3* conditional allele, which produces a wildtype band of ~250bp and a mutant band of ~350bp, is: 5’GCGTCTTCATGTGAATGATGGTG3’ and 5’GACCAAAAGCAATCTCTTGGTTCTAC3’.

For qRT-PCR, total RNA was isolated by TRIzol® Reagent (Invitrogen) and cDNA was synthesized using Verso^TM^ Reverse Transcriptase (Thermo Scientific). Real time PCR was performed on Applied Biosystems 7300 Real-Time PCR System using GoTaq® qPCR Master Mix (Promega). Expression levels of target genes were normalized to GAPDH level using the 2^−Δ Ct^ method. Relative gene expression levels were determined by normalizing wild type gene expression level to 100%. Primer sequences for real time PCR are:

GAPDH: forward 5’- TGCCCCCATGTTTGTGATG-3’, reverse 5’- TGTGGTCATGAGCCCTTCC-3’;

Wnt7a: forward 5’- GGCTCCCAGACAGCGGGCAA-3’, reverse 5’- CGGAACTGAAACTGACAC-3’;

Wnt7b: forward 5’- TCTCTGCTTTGGCGTCCTCTAC-3’, reverse 5’- GCCAGGCCAGGAATCTTGTTG-3’;

Sfrp1: forward 5’-TCAGAGGCCATCATTGAACA-3’, reverse 5’- CCCAGCTTCAAGGGTTTCTT-3’;

Sfrp2: forward 5’- ATGGAAACCCTTTGTAAAAATGACT-3’, reverse 5’- TCTTGCTCTTTGTCTCCAGGATGAT-3’;

Wif1: forward 5’- CCACCTGAGGAGAGCTTGTACC-3’, reverse 5’- TGGCATTCTTTGTTGGGCTTTCC-3’;

Dkk1: forward 5′-CCGGGAACTACTGCA AAAAT-3′, reverse 5′-GGTTTTCAATGATGCTTTCCTC-3′;

Agpt1: forward 5′- CTACCAACAACAACAGCATCC-3′, reverse 5′-CTCCCTTTAGCAAAACACCTTC-3′;

VEGF: forward (common) 5’- GCCAGCACATAGGAGAGATGAGC -3’

VEGF120: reverse 5’- GGCTTGTCACATTTTTCTGG-3′;

VEGF164: reverse 5’- CAAGGCTCACAGTGATTTTCTGG-3′;

VEGF188: reverse 5’- AACAAGGCTCACAGTGAACGCT-3′.

Immunohistochemistry

The following primary antibodies were used at respective dilutions/concentrations: mouse anti-BrdU supernatant (clone G3G4, Developmental Studies Hybridoma Bank (DSHB), University of Iowa, IA; 1:40), mouse anti-Nestin supernatant (DSHB; 1:20), mouse anti-RC2 supernatant (DSHB; 1:10), moue anti-GLAST (ACSA-1, Miltenyi Biotec, 1:50), rabbit anti-BLBP (Millipore: 1:400), rat anti-EOMES (Tbr2) (Dan11mag, eBioscience, 1:500), rat anti-Ctip2 (Abcam, 1:500), rabbit anti-phospho Histone H3 (Ser10) (Millipore; 1:400), rabbit anti-Tbr1 (Millipore; 1:300), rabbit anti-Cux1 (CDP) (Santa Cruz; 1:100), rabbit anti-laminin (Sigma; 1:2000), goat anti-MMP2 (R&D Systems; 5 μg/ml), rabbit anti-Glut-1 (Thermo Scientific; 1:200), rabbit anti-NG2 (Millipore, 1:400), rabbit anti-LEF1 (AVIVA;1:100), rabbit anti-Ki67 Ab-4 (Thermo Scientific; 1:100), rabbit anti-Desmin (Millipore; 1:500), mouse anti-CD31 (PECAM-1) supernatant (clone 2H8, DSHB; 1:10), rat anti-CD31 (BD Pharmingen, 1:100), rat anti-CD11b (BD Pharmingen, 1:200), rat anti-Ter119 (BD Pharmingen, 1:200), and biotinylated IB4 (Sigma; 20 μg/ml). FITC and Cy3 conjugated secondary antibodies were purchased from Jackson ImmunoResearch Laboratories (West Grove, PA). Peroxidase conjugated secondary antibodies were purchased from Santa Cruz Biotech. Staining procedures were performed as described previously (Huang et al., 2006), and sections were mounted with ProLong Antifade medium (Invitrogen Molecular Probes, Carlsbad, CA) and analyzed under a Nikon *eclipse* Ti microscope. For 3-D reconstruction, Z-stack images were collected under an Olympus confocal microscope and processed using ImageJ software.

Cortical cell culture

Cortices were dissected from BAT-Gal reporter mice at E15.5 and E13.5. Tissues were mechanically dissociated and cells were re-suspended in 1 ml DMEM (Invitrogen) containing 10% fetal bovine serum (FBS) (Sigma) after centrifugation at 1,000 rpm for 5min. 3-5 million cells per well were then incubated at 37°C overnight on coverslips pre-coated with 50 µg/ml poly-D-lysine and 50 µg/ml laminin. Coverslips were gently rinsed with 1xDPBS three times and fixed with 2% PFA containing 0.2% gluteraldehyde (Electron Microscopy Sciences) for 5min on ice. Fixed cells were stained for lacZ expression by immersing coverslips in X-gal staining solution at 37°C overnight. After washing, coverslips were then set up for immunocytochemistry using anti-BLBP (Millipore) and anti-PECAM (CD31) (BD Pharmingen) primary antibodies at 4°C overnight. Secondary antibodies were applied at room temperature for 4 hours. Cells were analyzed from 25-30 fields per coverslip from at least three independent experiments for each condition. PECAM and BLBP positive cells were identified by immunofluorescence while X-gal staining was analyzed under bright field.
